# Supplementary material for: Diversification and recurrent adaptation of the synaptonemal complex in Drosophila
Source: PLoS Genet. 2025 Jan 13;21(1):e1011549. doi: 10.1371/journal.pgen.1011549 (PMC11761671; doi:10.1371/journal.pgen.1011549)
Supplement: S2 Fig — Dotplots of self-alignments of the 500kb regions on the neo-X (A) and neo-Y (B) containing ord. Repeated tandem duplications can be observed generating many alignments off the diagonal. (PDF) [file pgen.1011549.s005.pdf]

A D. miranda X-linked ord tandem copy regions: MullerC:12,770,000-12,830,000

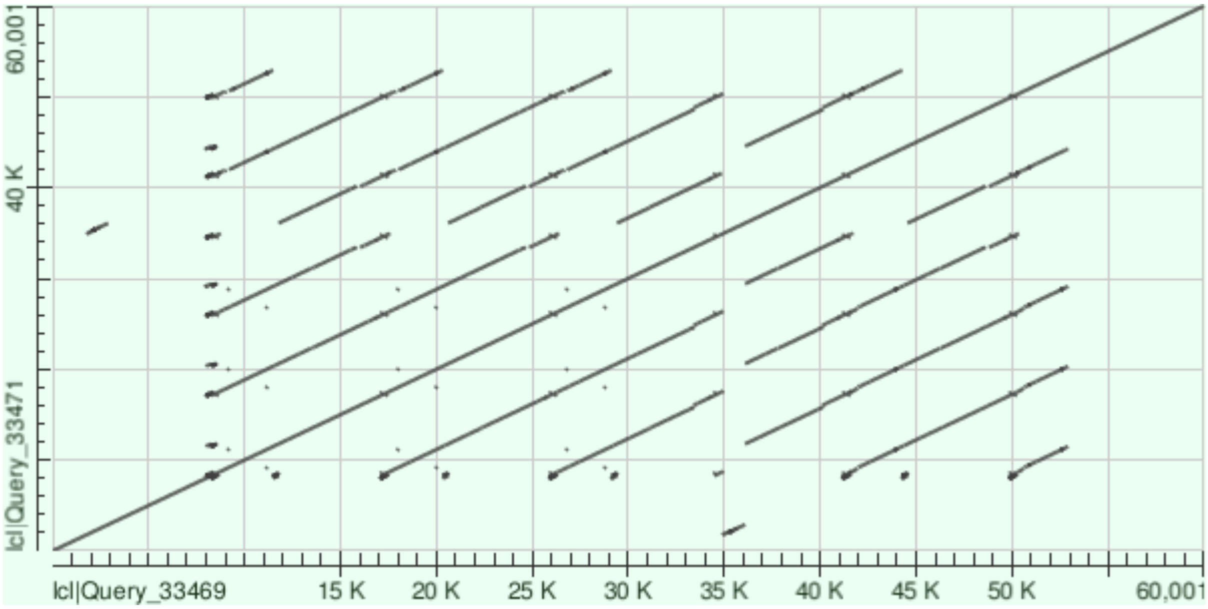

B D. miranda Y-linked ord region: Contig_Y1:27,200,000-27,700,000

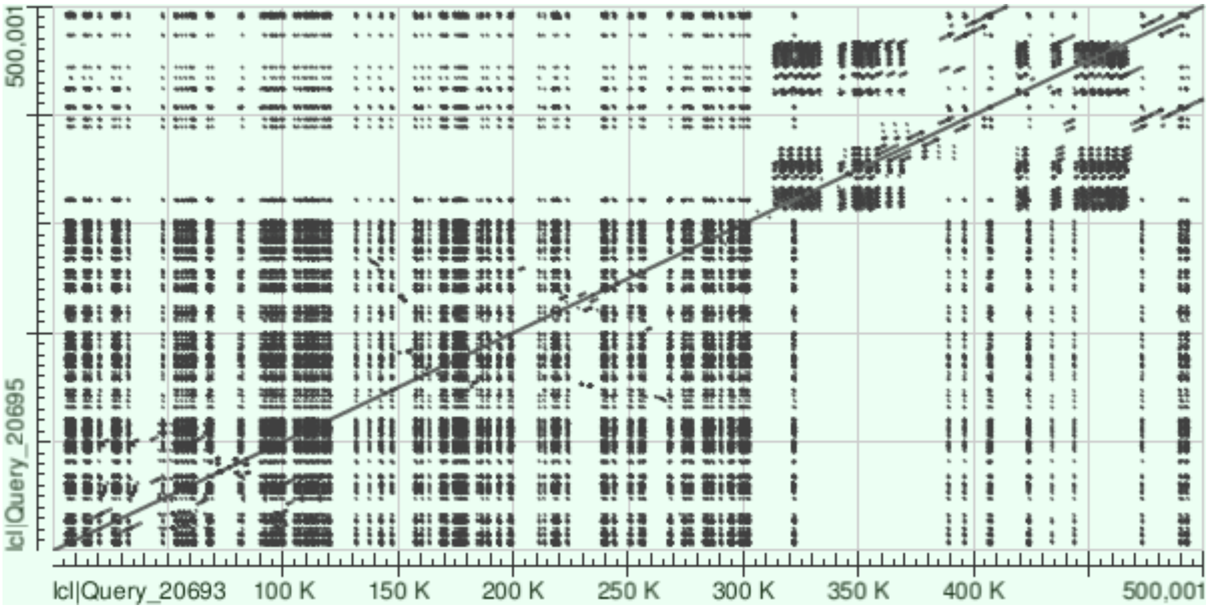

Supplementary Figure 2: *Dotplots of self-alignments of the 500kb regions on the neo-X (A) and neo-Y (B) containing ord. Repeated tandem duplications can be observed generating many alignments off the diagonal.*
