# Supplementary material for: Diversification and recurrent adaptation of the synaptonemal complex in Drosophila
Source: PLoS Genet. 2025 Jan 13;21(1):e1011549. doi: 10.1371/journal.pgen.1011549 (PMC11761671; doi:10.1371/journal.pgen.1011549)
Supplement: S4 Fig — Gene trees for c(2)M (A), ord (B), corolla (C) and cona (D-F) constructed using the protein alignments. Trees for cona are based on Prank (D), MUSCLE (E), and MAFFT (F) protein alignments. (PDF) [file pgen.1011549.s007.pdf]

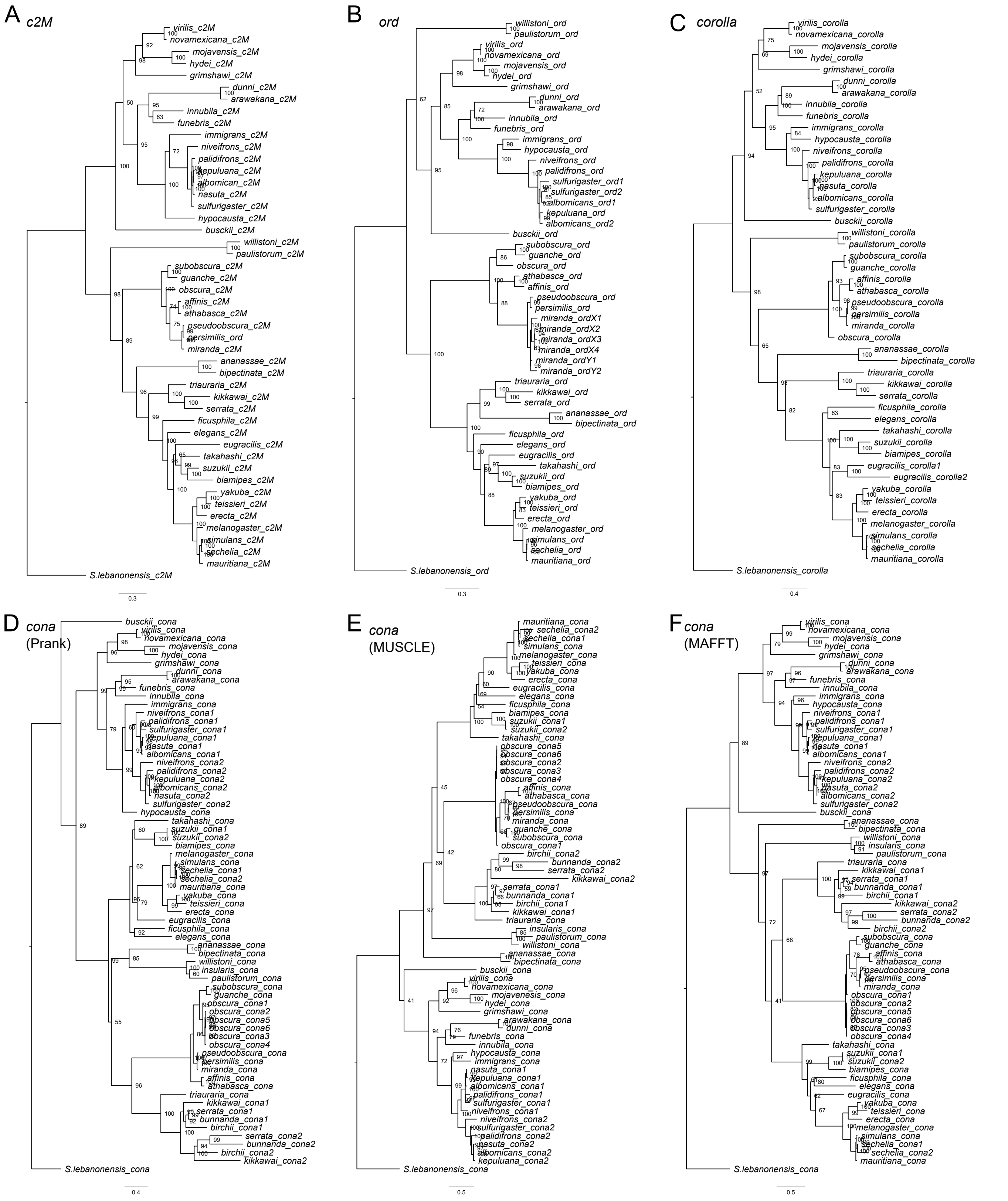

Supplementary Figure 4: Gene trees for c(2)M (A), ord (B), corolla (C) and cona (D-F) constructed using the protein alignments. Trees for cona are based on Prank (D), MUSCLE (E), and MAFFT (F) protein alignments.
