# Supplementary material for: Diversification and recurrent adaptation of the synaptonemal complex in Drosophila
Source: PLoS Genet. 2025 Jan 13;21(1):e1011549. doi: 10.1371/journal.pgen.1011549 (PMC11761671; doi:10.1371/journal.pgen.1011549)
Supplement: S5 Fig — 6 tandem duplicates of cona in D. obscura can be observed. (PDF) [file pgen.1011549.s008.pdf]

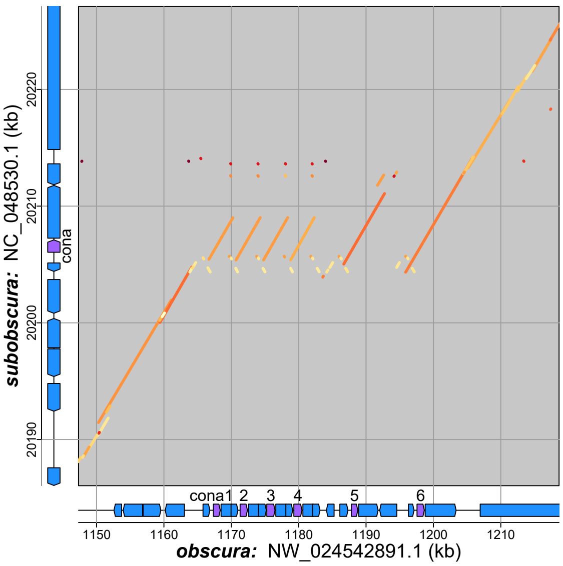

Supplementary Figure 5: Dotplot showing the alignment between subobscura and obscura. 6 tandem duplicates of cona in D. obscura can be observed..
