# Supplementary material for: Diversification and recurrent adaptation of the synaptonemal complex in Drosophila
Source: PLoS Genet. 2025 Jan 13;21(1):e1011549. doi: 10.1371/journal.pgen.1011549 (PMC11761671; doi:10.1371/journal.pgen.1011549)
Supplement: S6 Fig — Each arrow indicates a syntenic region where orthologs are found. Muller elements are labeled by different colors and are not drawn to scale. Order of the arrows do not reflect their relative chromosomal locations. Open arrow for corolla indicates insertion into repeat rich pericentromeric regions. B. Dotplot of the genomic region surround c(3)G paralogs in D. innubila compared to D. funebris. Phylogenetic reconstruction of c(3)G position and movements in the genome. Color of branches indicate the Muller elements in which c(3)G resides. Different patterns represent different, non-syntenic locations, on the Muller elements. Arrows point to the D. innubila duplicates which are found in ancestral and derived regions suggesting an old duplication prior to species split with D. funebris. (PDF) [file pgen.1011549.s009.pdf]

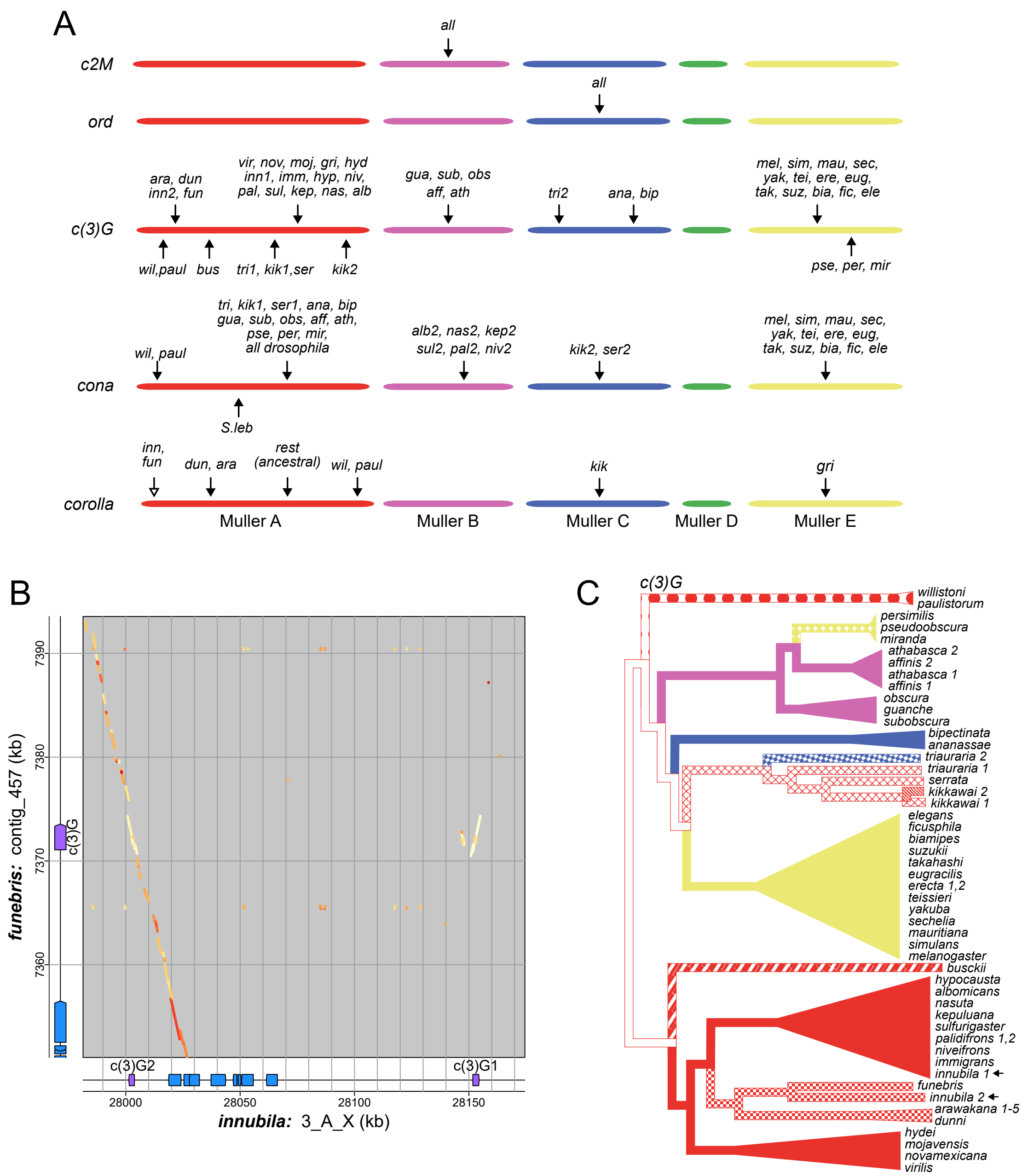

Supplementary Figure 6: Muller elements in which SC components are found are labeled. Each arrow indicates a syntenic region where orthologs are found. Muller elements are labeled by different colors and are not drawn to scale. Order of the arrows do not reflect their relative chromosomal locations. Open arrow for corolla indicates insertion into repeat rich pericentromeric regions. B. Dotplot of the genomic region surround c(3)G paralogs in D. innubila compared to D. funebris. Phylogenetic reconstruction of c(3)G position and movements in the genome. Color of branches indicate the Muller elements in which c(3)G resides. Different patterns represent different, non-syntenic locations, on the Muller elements. Arrows point to the D. innubila duplicates which are found in ancestral and derived regions suggesting an old duplication prior to species split with D. funebris.
