# Supplementary material for: Diversification and recurrent adaptation of the synaptonemal complex in Drosophila
Source: PLoS Genet. 2025 Jan 13;21(1):e1011549. doi: 10.1371/journal.pgen.1011549 (PMC11761671; doi:10.1371/journal.pgen.1011549)
Supplement: S7 Fig — A. Alignment of corolla CDS (center track) to the D. dunni (top track) and D.arawakana (bottom track) genomes. B. Self alignment of the genomic region containing corolla revealing complex tandem repeat structures. (PDF) [file pgen.1011549.s010.pdf]

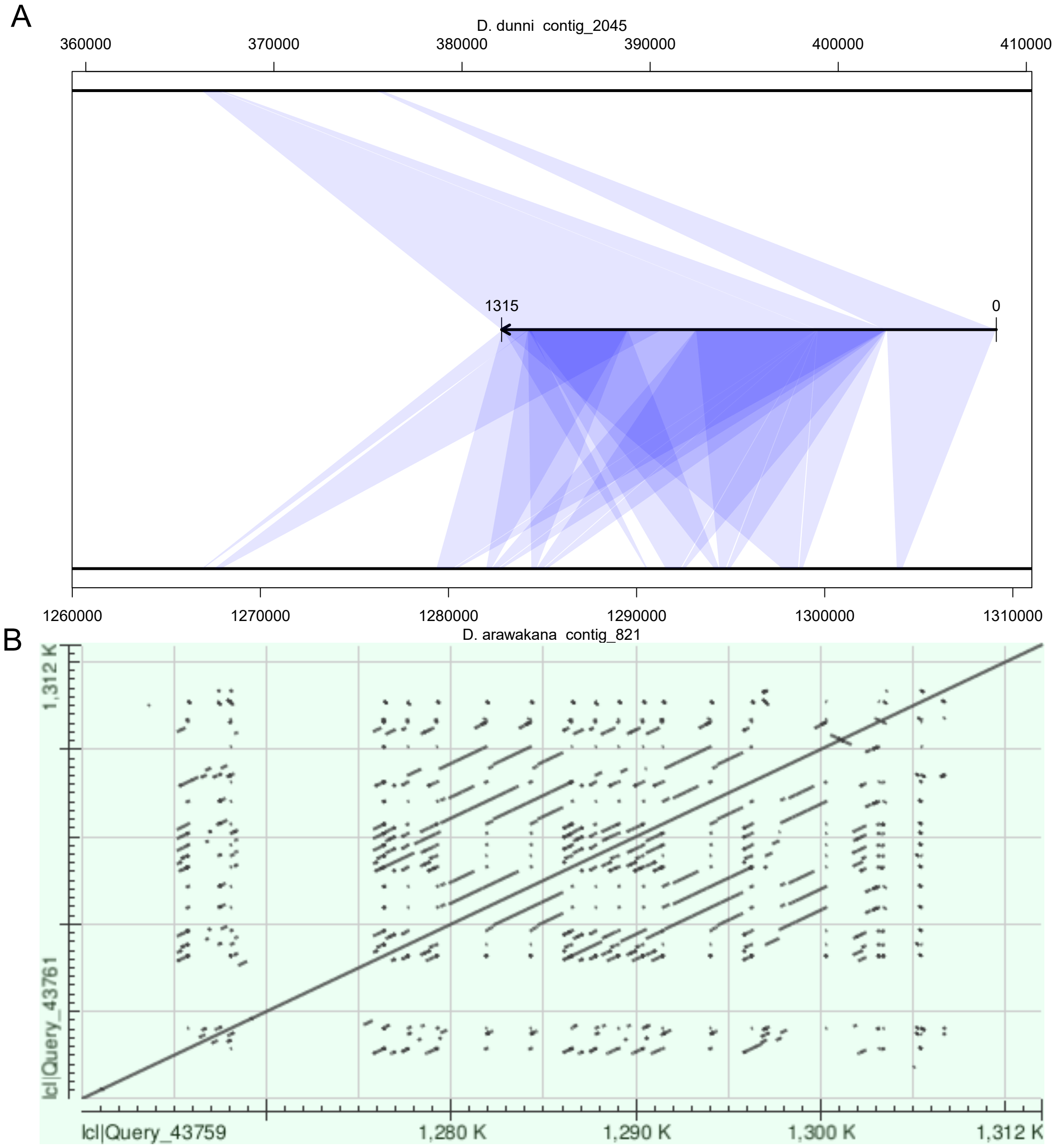

Supplementary Figure 7: Truncations of tandem copies of corolla in D. arawakana. A. Alignment of corolla CDS (center track) to the D. dunni (top track) and D.arawakana (bottom track) genomes. B. Self alignment of the genomic region containing corolla revealing complex tandem repeat structures.
