# Supplementary material for: Diversification and recurrent adaptation of the synaptonemal complex in Drosophila
Source: PLoS Genet. 2025 Jan 13;21(1):e1011549. doi: 10.1371/journal.pgen.1011549 (PMC11761671; doi:10.1371/journal.pgen.1011549)
Supplement: S8 Fig — A. c(3)G moved to Muller E in the pseudoobscura subgroup. Blastn alignment between the c(3)G location in D. miranda to the syntenic region in D. affinis which lacks c(3)G. c(3)G and flanking genes are boxed in red. B. Self alignment of the region c(3)G migrated to show extensive tandem repeat structure in D. affinis. Region where c(3)G inserted into boxed in red. C. Self alignment of the syntenic region in D. obscura. (PDF) [file pgen.1011549.s011.pdf]

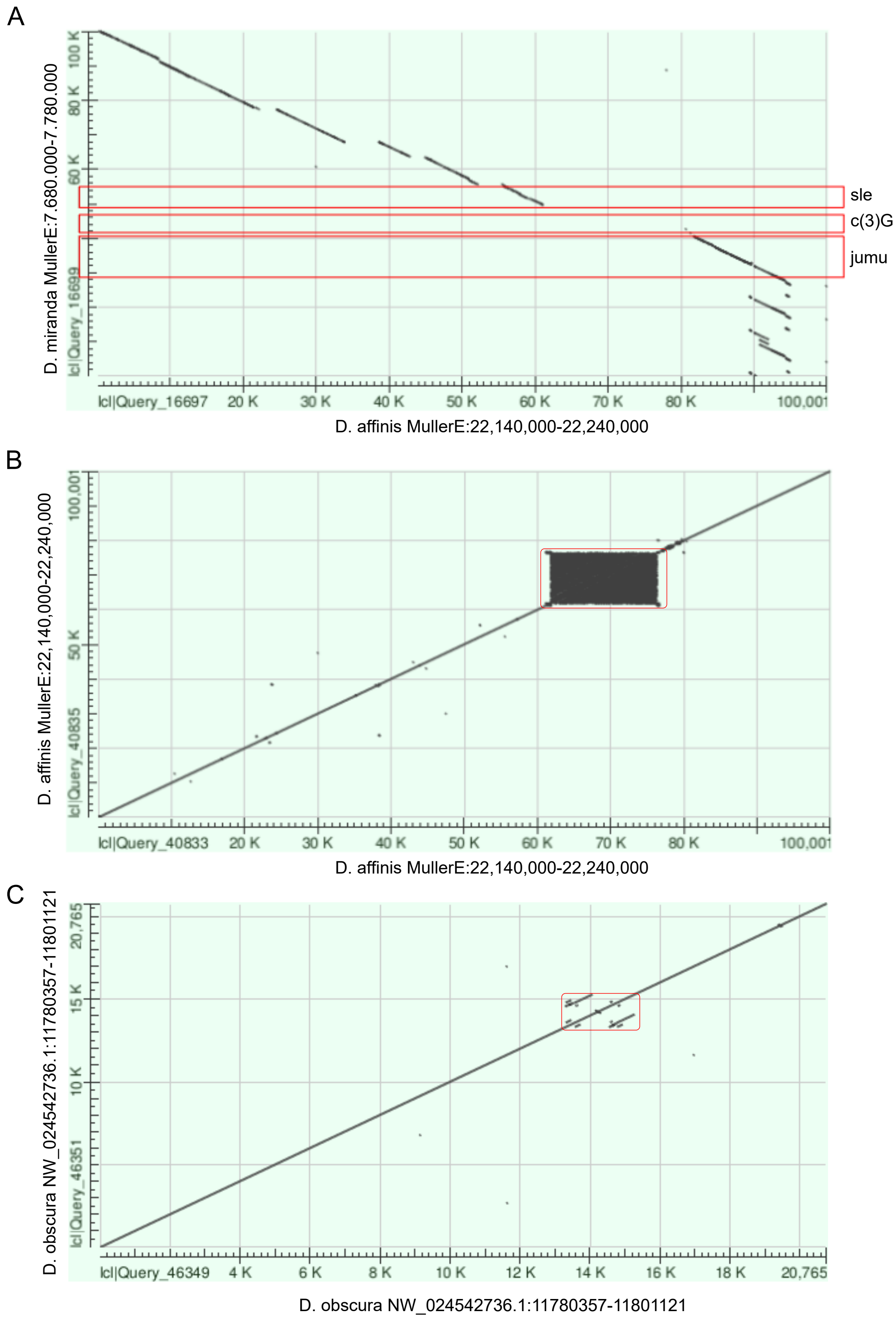

Supplementary Figure 8: c(3)G movement in the pseudoobscura subgroup. A. c(3)G moved to Muller E in the pseudoobscura subgroup. Blastn alignment between the c(3)G location in D. miranda to the syntenic region in D. affinis which lacks c(3)G. c(3)G and flanking genes are boxed in red. B. Self alignment of the region c(3)G migrated to show extensive tandem repeat structure in D. affinis. Region where c(3)G inserted into boxed in red. C. Self alignment of the syntenic region in D. obscura.
