# Supplementary figures and images for: Diversification and recurrent adaptation of the synaptonemal complex in Drosophila
Source: PLoS Genet. 2025 Jan 13;21(1):e1011549. doi: 10.1371/journal.pgen.1011549 (PMC11761671; doi:10.1371/journal.pgen.1011549)

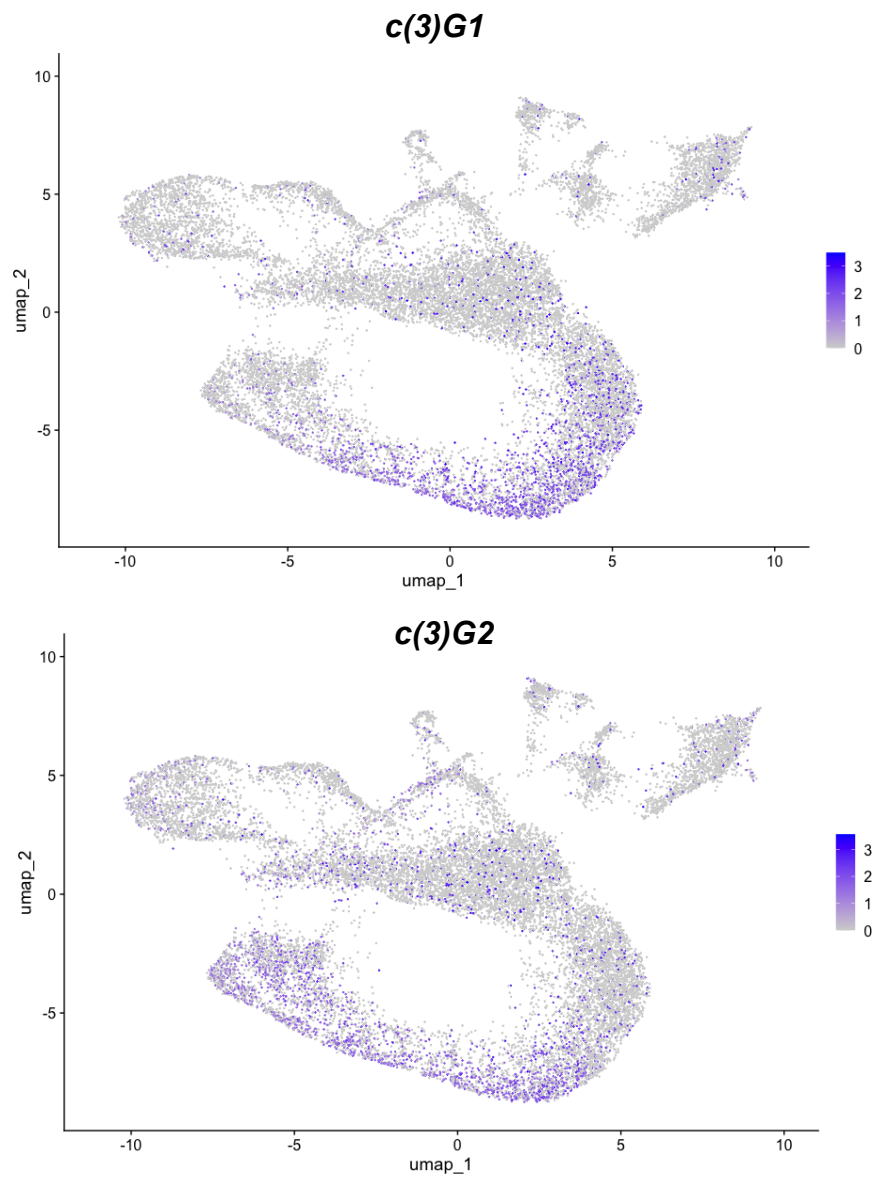

**Supplementary Figure 10:** Expression of c(3)G1 and c(3)G2 in *D. affinis* single nuclei RNA-seq dataset.

Supplement: S10 Fig — (PDF) [file pgen.1011549.s013.pdf]

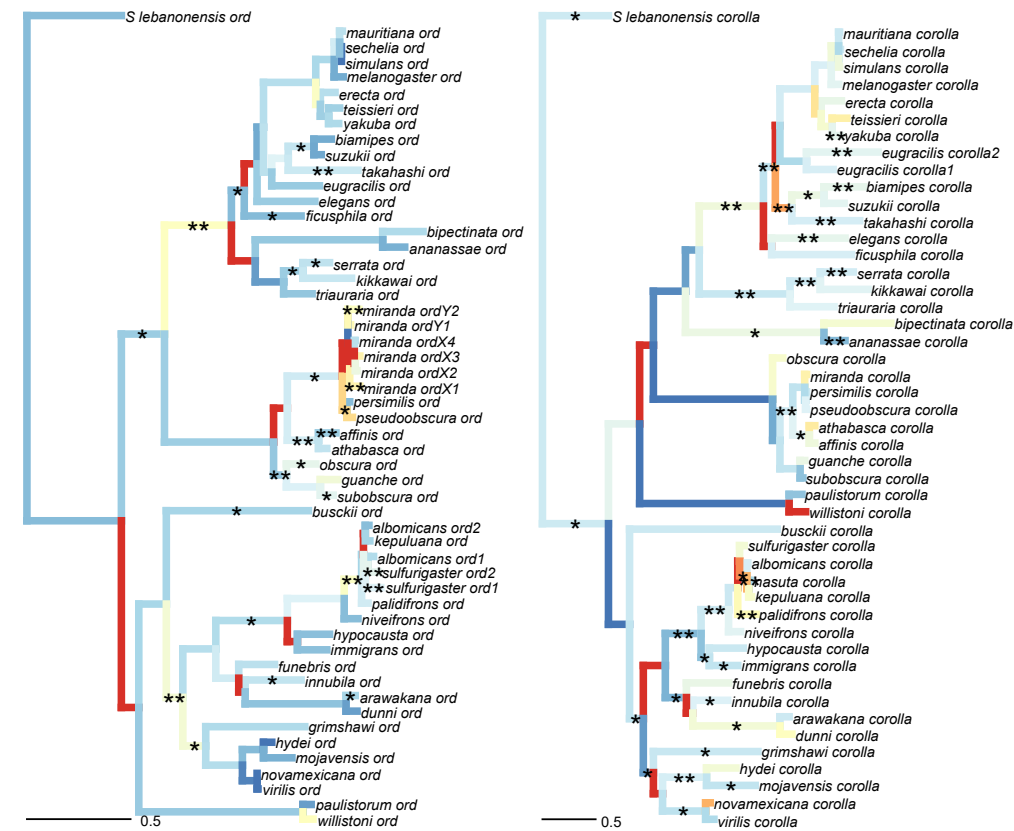

**Supplementary Figure 13: Branch specific Ka/Ks for ord, corolla, and cona**

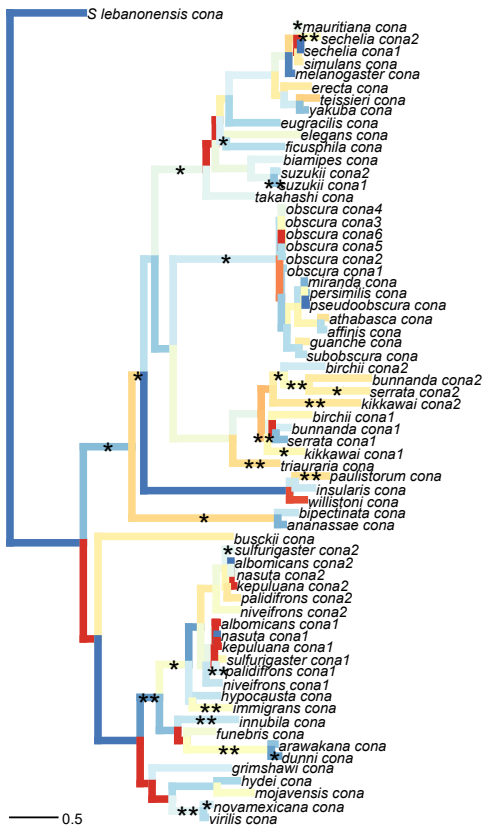

Supplement: S13 Fig — (PDF) [file pgen.1011549.s016.pdf]
