# Supplementary material for: Diversification and recurrent adaptation of the synaptonemal complex in Drosophila
Source: PLoS Genet. 2025 Jan 13;21(1):e1011549. doi: 10.1371/journal.pgen.1011549 (PMC11761671; doi:10.1371/journal.pgen.1011549)
Supplement: S11 Fig — Pink and purple differentiate reads originating from the sense and antisense strands. Note the genome track is in the reverse orientation compared to Fig 3D. (PDF) [file pgen.1011549.s014.pdf]

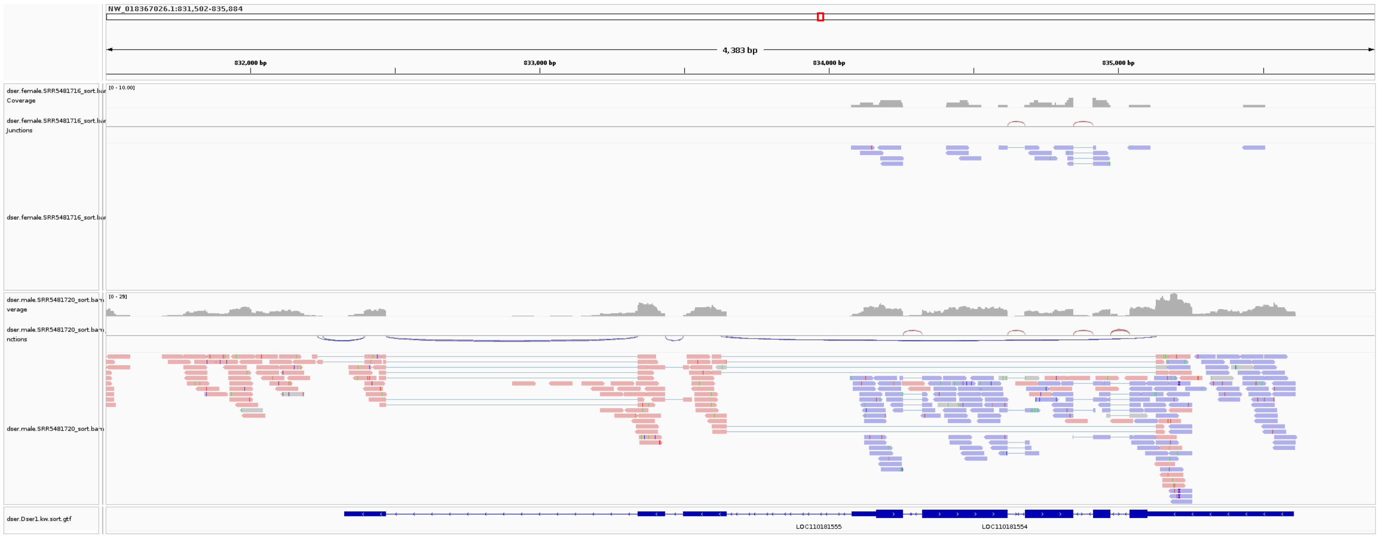

Supplementary Figure 11: IGV genome tracks of D. serrata showing expression surrounding cona2 (marked by purple reads) and the anti-sense lncRNA (marked by pink reads). Pink and purple differentiate reads originating from the sense and antisense strands. Note the genome track is in the reverse orientation compared to Figure 3D.
