# Supplementary material for: Diversification and recurrent adaptation of the synaptonemal complex in Drosophila
Source: PLoS Genet. 2025 Jan 13;21(1):e1011549. doi: 10.1371/journal.pgen.1011549 (PMC11761671; doi:10.1371/journal.pgen.1011549)
Supplement: S12 Fig — It is embedded in the intron of the gene teiresias. Note the lack of ortholog in the gray track. (PDF) [file pgen.1011549.s015.pdf]

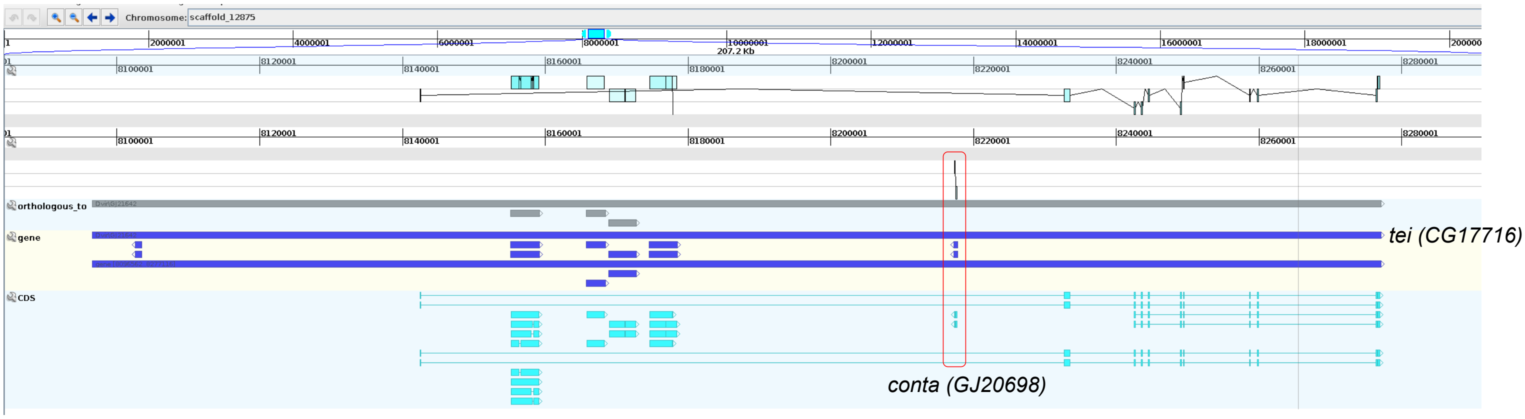

Supplementary Figure 12: *conta* location in the *D. virilis* genome. It is embedded in the intron of the gene *teiresias*. Note the lack of ortholog in the gray track.
