# Supplementary material for: Diversification and recurrent adaptation of the synaptonemal complex in Drosophila
Source: PLoS Genet. 2025 Jan 13;21(1):e1011549. doi: 10.1371/journal.pgen.1011549 (PMC11761671; doi:10.1371/journal.pgen.1011549)
Supplement: S14 Fig — UMAP projection of testes cell types. Relevant cell types are labeled. (PDF) [file pgen.1011549.s017.pdf]

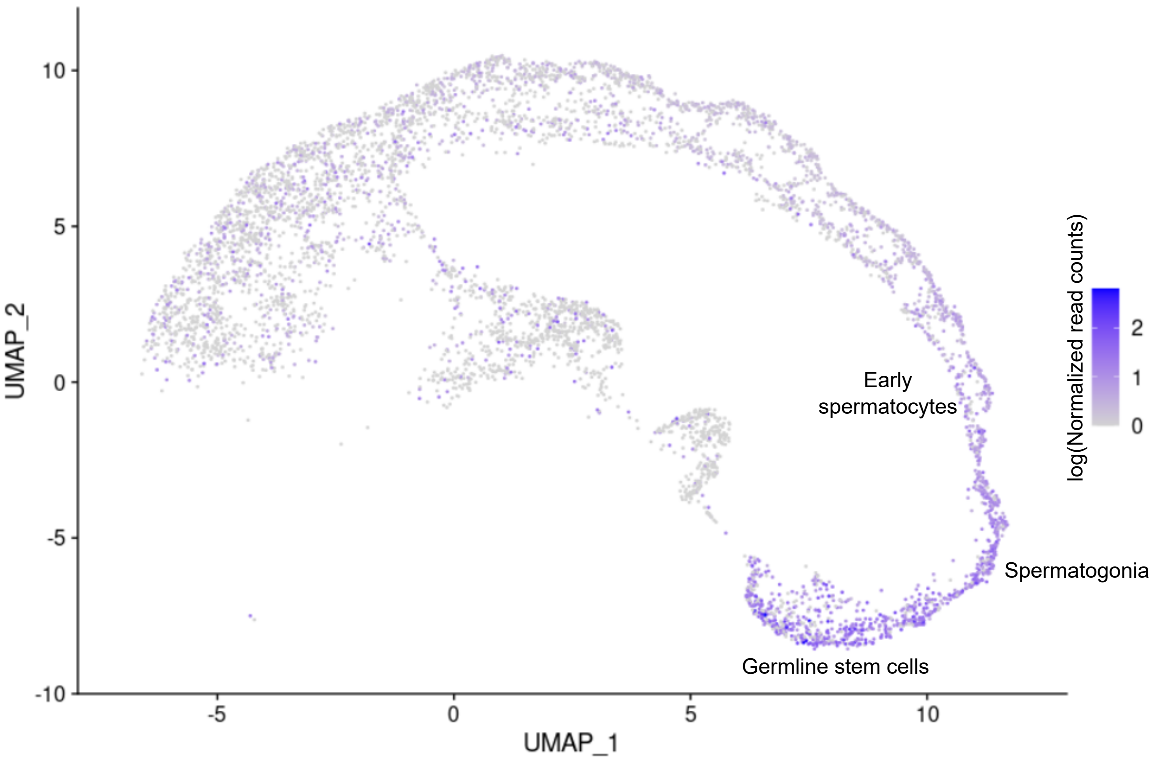

Supplementary Figure 14: Expression of c(3)G in testes single cell RNA-seq data for D. miranda. UMAP projection of testes cell types. Relevant cell types are labeled.
