# Supplementary material for: Diversification and recurrent adaptation of the synaptonemal complex in Drosophila
Source: PLoS Genet. 2025 Jan 13;21(1):e1011549. doi: 10.1371/journal.pgen.1011549 (PMC11761671; doi:10.1371/journal.pgen.1011549)
Supplement: S15 Fig — A. Ovary expression. P-value of pairwise Brown Forsythe test of equal variance for ovary expssion (B) and testes expression (C). (PDF) [file pgen.1011549.s018.pdf]

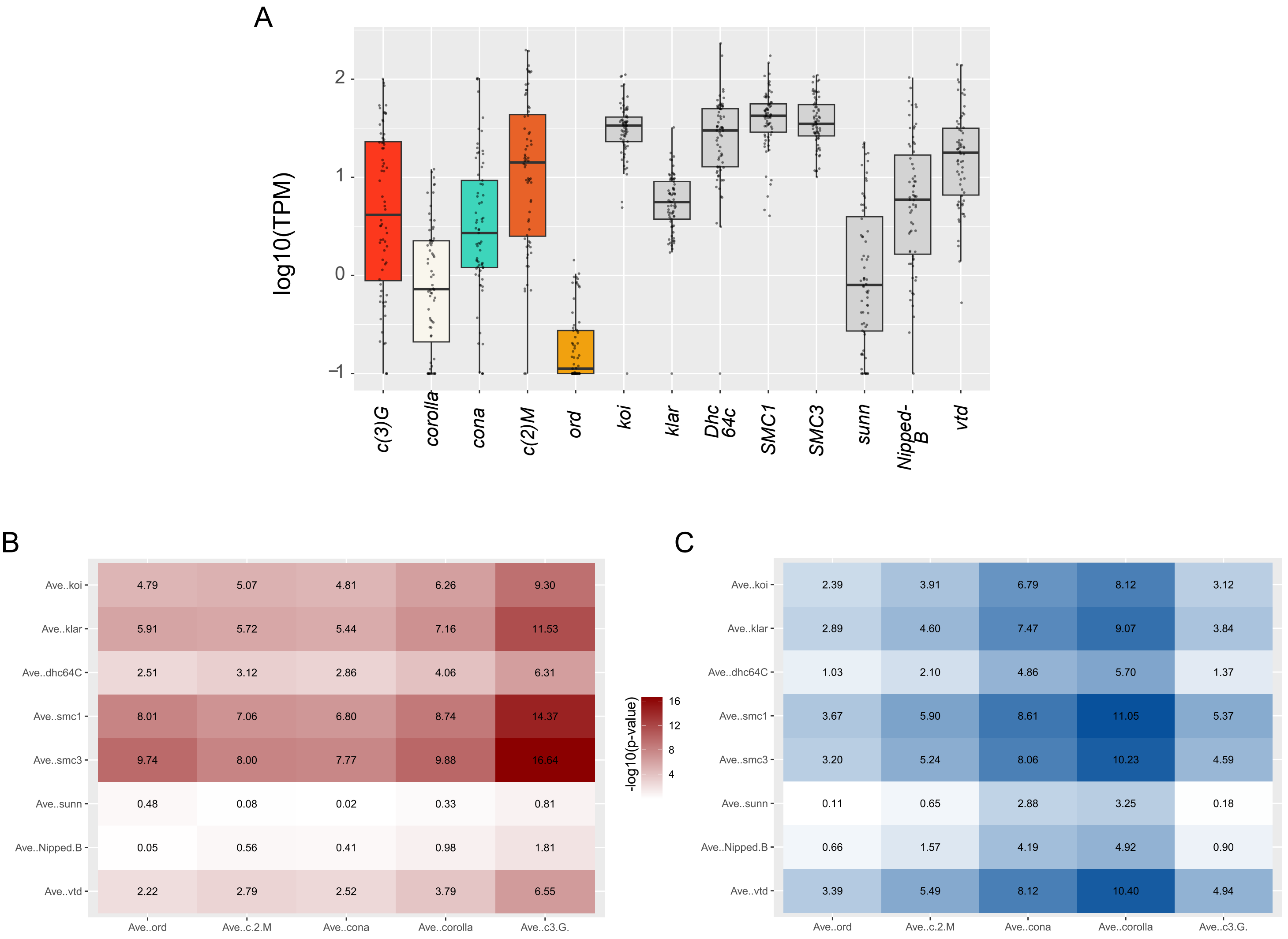

Supplementary Figure 15: Gene expression of SC and pairing genes in different *Drosophila* species. A. Ovary expression. P-value of pairwise Brown Forsythe test of equal variance for ovary expsion (B) and testes expression (C).
