# Supplementary material for: Diversification and recurrent adaptation of the synaptonemal complex in Drosophila
Source: PLoS Genet. 2025 Jan 13;21(1):e1011549. doi: 10.1371/journal.pgen.1011549 (PMC11761671; doi:10.1371/journal.pgen.1011549)
Supplement: S16 Fig — Expression of SC genes in different temperatures in D. melanogaster ovaries (A), D melanogaster males whole bodies (B), and D. suzukii testes (C). (PDF) [file pgen.1011549.s019.pdf]

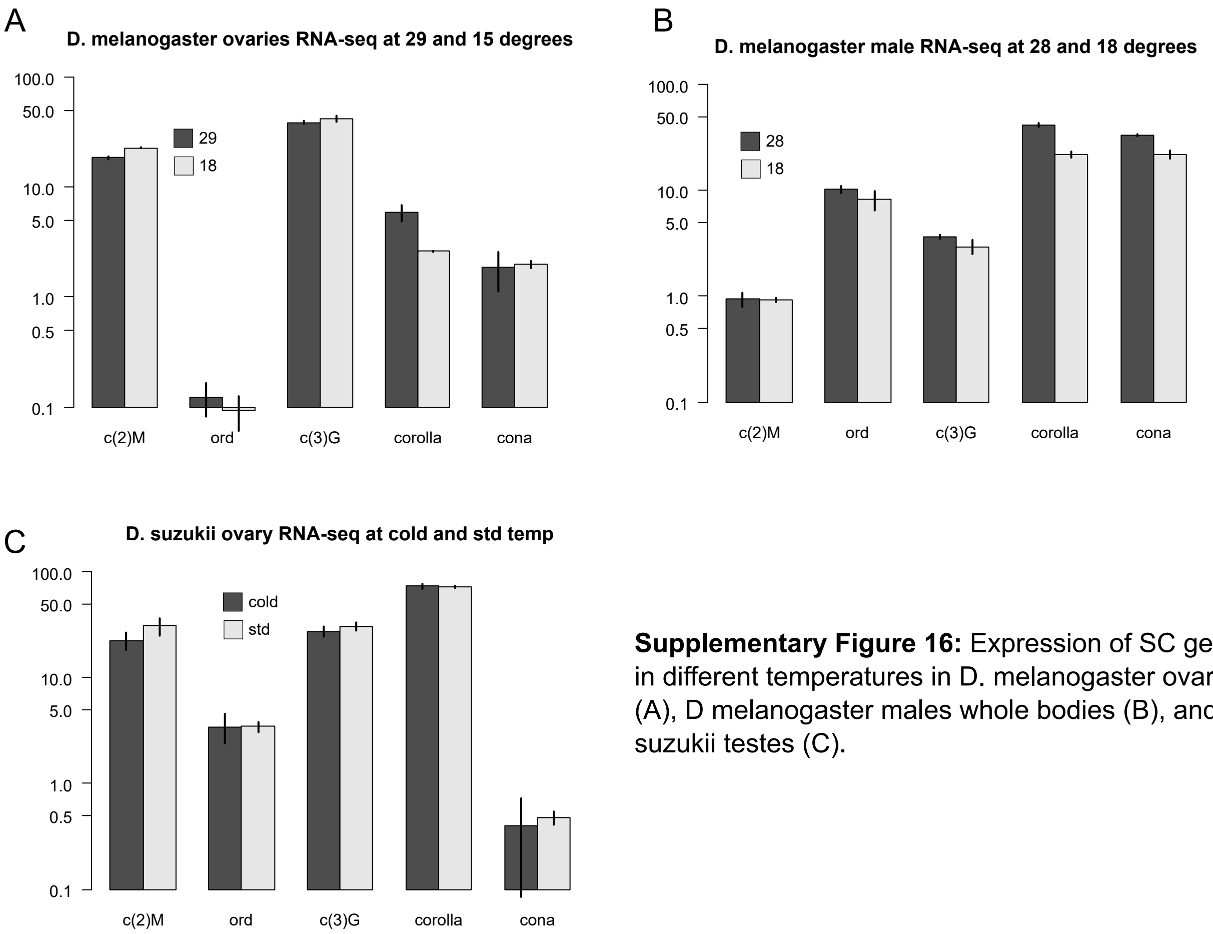

Supplementary Figure 16: Expression of SC genes in different temperatures in D. melanogaster ovaries (A), D melanogaster males whole bodies (B), and D. suzukii testes (C).
