# Supplementary material for: Diversification and recurrent adaptation of the synaptonemal complex in Drosophila
Source: PLoS Genet. 2025 Jan 13;21(1):e1011549. doi: 10.1371/journal.pgen.1011549 (PMC11761671; doi:10.1371/journal.pgen.1011549)
Supplement: S17 Fig — Modified from the adult cell type atlas from Flybase. (PDF) [file pgen.1011549.s020.pdf]

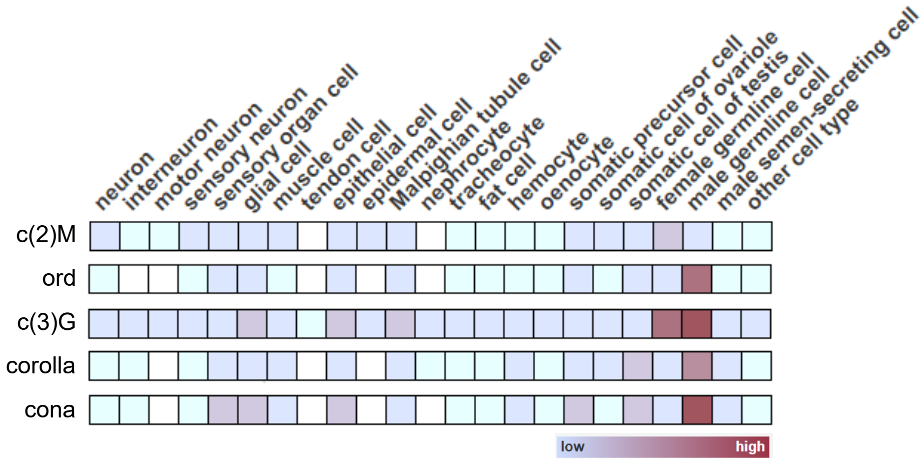

Supplementary Figure 17: SC gene expression across tissues. Modified from the adult cell type atlas from Flybase.
