# Supplementary material for: Diversification and recurrent adaptation of the synaptonemal complex in Drosophila
Source: PLoS Genet. 2025 Jan 13;21(1):e1011549. doi: 10.1371/journal.pgen.1011549 (PMC11761671; doi:10.1371/journal.pgen.1011549)
Supplement: S19 Fig — Qunatile quantile plot of the observed and expected p-value (in -log10 scale) for the rapidly evolving c(3)G and conserved vtd shows that in both cases the reported p-values are not sensitive especially when p-values are high. Despite this, c(3)G clearly deviates from expected due to elevated rates of protein evolution, but multiple testing correction removes large number of the nominally significant data points. (PDF) [file pgen.1011549.s022.pdf]

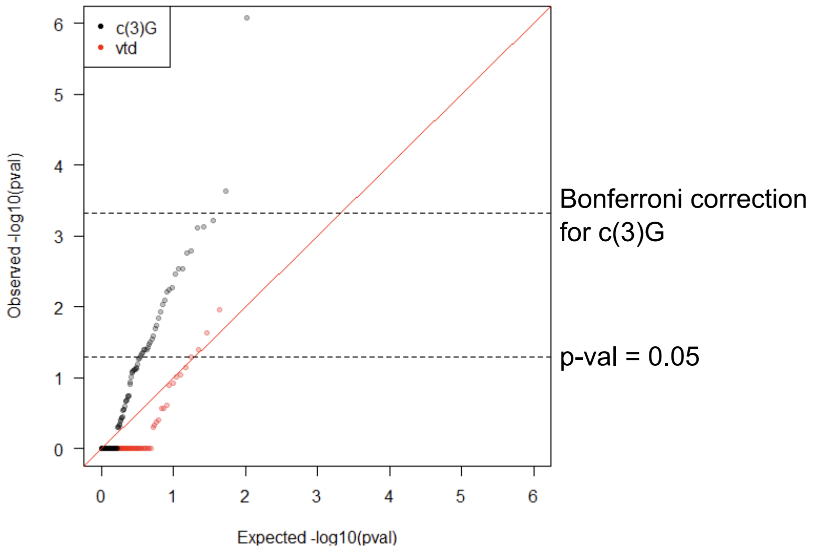

Supplementary Figure 19: Power analysis of Hyphy's statistical procedure to detect significant positive selection. Qunatile quantile plot of the observed and expected p-value (in -log10 scale) for the rapidly evolving *c(3)G* and conserved *vtd* shows that in both cases the reported p-values are not sensitive especially when p-values are high. Despite this, c(3)G clearly deviates from expected due to elevated rates of protein evolution, but multiple testing correction removes large number of the nominally significant data points.
