# Supplementary material for: Diversification and recurrent adaptation of the synaptonemal complex in Drosophila
Source: PLoS Genet. 2025 Jan 13;21(1):e1011549. doi: 10.1371/journal.pgen.1011549 (PMC11761671; doi:10.1371/journal.pgen.1011549)
Supplement: S3 Fig — Pairwise % identity values above and below the diagonal were estimated based on blastn of the CDS and blastp of the AA sequences, respectively. (PDF) [file pgen.1011549.s006.pdf]

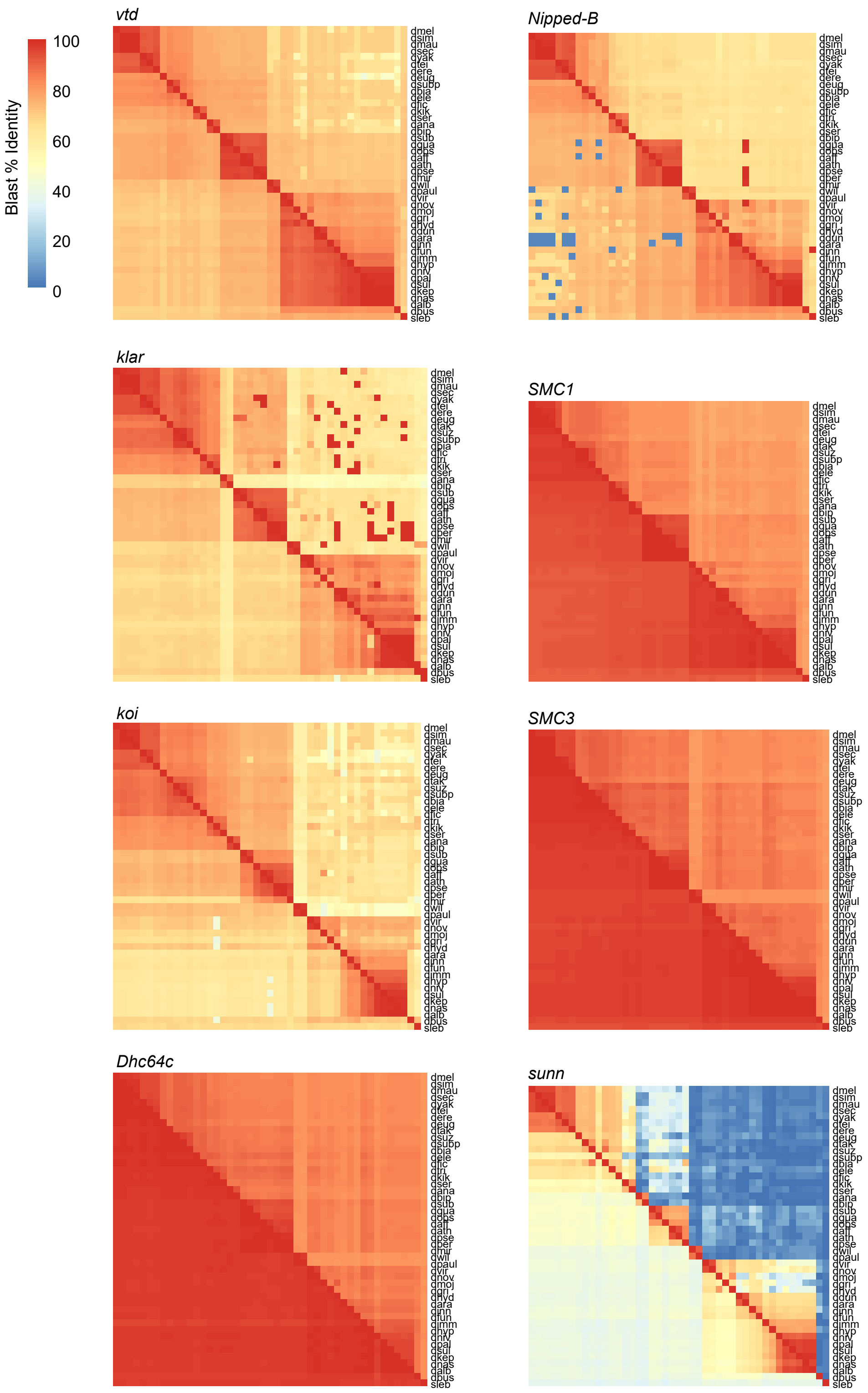

Supplementary Figure 3: Sequence similarity of orthologs of genes involved in meiotic pairing during female meiosis as measured by blast (same as Figure 1D-F). Pairwise % identity values above and below the diagonal were estimated based on blastn of the CDS and blastp of the AA sequences, respectively.
