# Supplementary material for: Diversification and recurrent adaptation of the synaptonemal complex in Drosophila
Source: PLoS Genet. 2025 Jan 13;21(1):e1011549. doi: 10.1371/journal.pgen.1011549 (PMC11761671; doi:10.1371/journal.pgen.1011549)
Supplement: Attachment — Submitted filename: Zakerzade_etal_Review.pdf [file pgen.1011549.s023.pdf]

Zakerzade et al. present a study exploring evolutionary dynamics of the synaptonemal complex in Drosophila. Some of these analyses have been done before on a small subset of species. This paper takes advantage of the recent release of high-quality Drosophila genomes and transcriptomes from closely related species across the phylogeny to comprehensively examine sequence evolution, gene duplication, and expression evolution of SC proteins. Of note, Zakerzade et al. reveal a striking signal of recurrent duplication and male-biased expression of SC genes, even though the synaptonemal complex does not form in the testes.

Major Comments:

In general, I found the manuscript a bit long. Below I suggest a few places that can be cut or shortened particularly merging a few of the results sections and focusing the discussion a bit more.

I would love the authors to strengthen the discussion and speculate more or even present a model about why SC genes in Drosophila may undergo recurrent diversification, particularly in the male germline. What might SC genes, particularly young duplicates, be doing in the male germline if not making a synaptonemal complex? The authors briefly mention meiotic conflict. It would greatly increase the novelty of the paper if the authors could speculate on a model of how SC genes could play a role in these arms races.

Perhaps related to my point above, the genes that comprise the SC central region show increased sequence divergence and duplication rates compared to the genes comprising the lateral region. I find this interesting since the lateral region genes touch chromatin while it seems the central region genes do not interact directly with DNA. Given this biology, could the authors speculate on the selective agent driving diversification of central region genes and why/how it contributes to different patterns of adaptive evolution between the two SC components?

Below I outline a few minor comments that I think will help improve the clarity of manuscript.

Minor Comments:

- I got confused between the central elements and the central region—which seems to consist of both central elements and transverse elements. It would be helpful to be more explicit about this when first introducing the central region components at Line 93.
- The molecular evolutionary analyses were conducted across the Drosophila phylogeny and across key species groups. The combination of the broad-scale and fine-scale analyses is great. It would be helpful to note how many "evolutionary years" these different groups encompass.
- Figure 1: The Blast% plots are very interesting. I would rather see this analysis for all five SC genes in the main text and move the Blast% for vtd to the Supplement with the other pairing genes. I understand that the authors are trying to contrast the striking divergence of the SC genes to other pairing genes, however I think this is effectively done in panel G showing pairwise Blastp % identity. I understand that moving the 3 additional Blast% heatplots might be challenging in terms of space. In general, I think components of Figure 1 can be smaller without sacrificing clarity, particularly panel A, the width of the panel B phylogenetic tree, and the Blast% heatplots could be smaller. Additionally, due to its placement, I initially thought that the Blast % identity scalebar was specific to panel E instead of to all the heatplots. Perhaps this scale can be moved to the top? Finally, what are the numbers in parentheses in panel G?
- Line 129: I understand the authors definition of malformed in text. In Figure 1, the authors represent "malformed" SC genes with a grey box, but all also have a blue symbol indicating a

copy. Does this mean that the SC genes in these species are still malformed? Or were they previously malformed but fixed in this paper by hand-annotation? Can the authors better clarify?

- Figure 2: The authors note that: “unless connected by lines, separate blocks of the same color represent different locations of the same element”. I found this notation a bit subtle for such an important result. This finding should be better represented in the text and in the figure. To emphasize this finding, I would rather see Supplementary Figure 6A in this main text figure instead of the dotplots. In general, dotplots might be hard for a broad reader to interpret, I would favor moving them to the Supplement.
- Line 152 and below is about duplication events. This text should be moved to the section below that discusses repeated duplications and paralogs.
- Figure 3: I find the depictions of the Alpha-fold structures to be a bit cramped making it hard to distinguish which regions correspond to different species.
- I understand that Kursel et al. showed that Caenorhabditis SC proteins evolve neutrally, but without reading the paper, this is a nuanced point in the face of “structural but not sequence conservation”. I wonder if this finding is better communicated to a naïve reader by noting instead that Caenorhabditis SC proteins have conserved coiled-coil domains and near invariant protein length but are among the most diverged sequences in Caenorhabditis.
- The convergent c(3)G duplications in D. athabasca/D. affinis and D. triauraria is so striking. This data needs a slower introduction to drive home this data. In particular, the D. athabasca/D. affinis and D. triauraria duplications are independent and therefore, should not both be named the same. Instead of naming both c(3)G2, the authors should choose separate identifiers.
- Do you know which two cell populations the two D. affinis c(3)G duplicates are expressed in (see Line 237)
- Independent cona duplicates in D. sechellia and D. serrata should also have different identifiers (see comment above)
- The section on coronetta, while interesting, is a bit long. I suggest that the authors trim this section by removing some of the extraneous details from Hemmer & Blumenstiel and merging a shortened version of this section into the previous section that outlines novel germline functions.
- Line 289: gene-wide omega values are predominantly negative—do the authors mean close to zero?
- Figure 5: Is it possible to match the order of the species in Panels A and B to the order of the species in Figure 2?
- How does the expression of SC genes in D. miranda from single cell RNA-seq data compare to that in D. melanogaster? Do these also have pre-meiotic activity? Presumably Li Zhao’s data set should be able to address this.
- The Discussion section “Trials & Tribulations can be shortened or entirely removed.
- Line 18: awkward sentence, might need a fix grammatically
- Line 26: awkward sentence, might need a fix grammatically
- Line 70: SC abbreviation here in introduction used before SC is defined at Line 76
- Lines 155: species spelled wrong
- Line 347: testes should be capitalized
- Line 450: subspecies spelled wrong
- D. sechellia spelled wrong throughout
