# Supplementary material for: Diversification and recurrent adaptation of the synaptonemal complex in Drosophila
Source: PLoS Genet. 2025 Jan 13;21(1):e1011549. doi: 10.1371/journal.pgen.1011549 (PMC11761671; doi:10.1371/journal.pgen.1011549)
Supplement: Attachment — Submitted filename: Response to reviewers.pdf [file pgen.1011549.s025.pdf]

We sincerely thank the reviewers and editor for their insightful comments and constructive feedback. With these comments and recommendations incorporated, we believe the manuscript is now substantially improved. Below is the point by point response to the questions and concerns.

Dear Dr. Wei,

Thank you very much for submitting your manuscript "Diversification and recurrent adaptation of the synaptonemal complex in Drosophila" (PGENETICS-D-24-00785) for consideration at PLOS Genetics. Your manuscript has been evaluated by three reviewers, all of whom recognized the novel and important contributions your team has made. However, all the reviewers also raised points that need to be addressed before your manuscript would be considered for publication. I have summarized below two main comments that were identified by more than one reviewer:

1. The manuscript reports that structural and expression evolution within synaptonemal complex genes is unusually rapid, but lacks explicit contrasts with other "control" gene sets to support this characterization. The comparison of Blastp % identity with genes involved in meiotic pairing is very informative regarding rates of protein evolution; can an analogous comparison be made to determine that the structural (gene duplication) and regulatory evolution of SC genes is also unusual? Given the effort and care that was required to uncover the patterns of SC gene evolution in this study, are the authors confident that similar evolutionary dynamism would not be discovered in other multi-protein complexes following such careful annotation across this phylogenetic range?
Given the unreliable state of gene annotations, we adopted two approaches to establish a control gene set or a genome-wide distribution. One is to curate an additional set of genes in a related process (meiotic pairing) as contrast. The other is to use large gene sets with published orthology calls to establish the genome-wide distribution albeit in fewer species. Now we have included them in our regulatory evolution analyses, showing that SC genes typically have higher expression divergence (with some exceptions) in both testes and ovaries (Figure 6B). Regarding the latter genome-wide sets, we previously only had rates of protein evolution for the gene set called by Clark et al 2007 on 6 species in the melanogaster species group, and showed that the SC genes have some of the highest rates of protein evolution. We now added analyses using orthodb calls on a restricted set of 15 species to evaluate the rate and distribution of regulatory evolution (Figure 6C). In this analysis, we also see that SC genes have rates of regulatory evolution at the higher end of the distribution (although not as extreme as protein-coding evolution) for both testis and ovary expression. The only exception is ord, which has consistently low expression in the ovaries.
For feasibility reasons, we are unable to check the extent of errors in these large datasets and databases. However, it stands to reason that better curation is more likely to decrease both the rates of protein and regulatory evolution. For example, better gene structure will

reduce alignment errors, inflating the false-positive rate in positive selection tests (Markova-Raina and Petrov 2011). Similarly chimeric and malformed gene structures will necessarily introduce more noise in gene expression estimates inflating the rate of regulatory divergence. Therefore, better curation will likely bring these distributions towards the low end. The SC genes - which we took the time to curate carefully - would then be even more strongly enriched for higher rates of evolution. We do not claim that such rates of evolution are exclusive to the SC, nor that other protein complexes cannot show such dynamic range. (In fact, the rate of regulatory evolution clearly shows that there are genes with higher rates in both testes and ovaries). But the message (and word of caution) here is that, in order to claim rapid evolution, one needs to make sure that there are no issues with the genomic data like annotation and assembly errors. Hence, we have the section “Trials and tribulations of gene ortholog and CDS searches from genome releases.”

2. Some of the manuscript’s conclusions assume gene function from gene expression data. This may be generally problematic, but is likely to be even more so for gene expression in reproductive tissues, particularly the testes. One concrete example is the disconnect between expression of SC genes and the phenotypes associated with mutant alleles of these genes in D. melanogaster. While this specific paradox is noted in the manuscript, the wider implications of inferring function from gene expression in species where no mutant data is available could be more carefully considered.
We have edited our manuscript to be more agnostic on the functional consequences of SC genes in the testes given gene expression. In many places, we change testes “function” to testes “activity” when appropriate. In the discussion section, we added a paragraph on the possibility of promiscuous expression in the testes and how it relates to our observations on activity and function in testes . We also noted in the discussion that lines of evidence support the possibility of functional consequences. For example, the careful study by Rubin et al 2022 identified subtle but interesting testes cellular phenotypes associated with SC mutants (c(3)G and cona) in melanogaster, despite the lack of clear, gross phenotype (Rubin et al. 2022). Similarly Kondo et al. 2017 found most new genes expressed in the testes contribute to male reproductive fitness (Kondo et al. 2017).

A revised manuscript should also consider individual reviewer comments. In particular, the discrepancies between the raw data and the results presented in the body of the manuscript, and the gaps in details of the PAML analyses that were noted by Reviewer 2 need to be addressed.
The discrepancy noted by reviewer 2 had to do with the use of adjusted vs nominal p-values. As stated, we elected to use the nominal p-values (due to an underpowered statistical procedure). We have now made this point more explicit in the manuscript by adding Table 1 which documents both nominal and adjusted p-values. We also now discuss in the methods and supplementary figure 19 the underpowered statistical procedure creating many false negatives. Some of the PAML analyses were previously captured in

Figure 5C. We have added supplementary table 4 for the PAML analyses about species groups.

Responses to reviewers questions

Reviewer #1:
Zakerzade et al. present a study exploring evolutionary dynamics of the synaptonemal complex in Drosophila. Some of these analyses have been done before on a small subset of species. This paper takes advantage of the recent release of high-quality Drosophila genomes and transcriptomes from closely related species across the phylogeny to comprehensively examine sequence evolution, gene duplication, and expression evolution of SC proteins. Of note, Zakerzade et al. reveal a striking signal of recurrent duplication and male-biased expression of SC genes, even though the synaptonemal complex does not form in the testes.

Major Comments:
In general, I found the manuscript a bit long. Below I suggest a few places that can be cut or shortened particularly merging a few of the results sections and focusing the discussion a bit more. I would love the authors to strengthen the discussion and speculate more or even present a model about why SC genes in Drosophila may undergo recurrent diversification, particularly in the male germline. What might SC genes, particularly young duplicates, be doing in the male germline if not making a synaptonemal complex? The authors briefly mention meiotic conflict. It would greatly increase the novelty of the paper if the authors could speculate on a model of how SC genes could play a role in these arms races.
We have trimmed some of the sections. However, since there's no word limit for research articles at PLoS Genetics, we elected to keep some of the sections mentioned for thoroughness and documentation.
We have expanded on the discussion regarding the testes function of SC genes and the possibility for meiotic conflict. We added a paragraph, albeit speculative, on potential sexual conflicts driving the repeated patterns of evolution we observed.

Perhaps related to my point above, the genes that comprise the SC central region show increased sequence divergence and duplication rates compared to the genes comprising the lateral region. I find this interesting since the lateral region genes touch chromatin while it seems the central region genes do not interact directly with DNA. Given this biology, could the authors speculate on the selective agent driving diversification of central region genes and why/how it contributes to different patterns of adaptive evolution between the two SC components?
This is a very interesting question, but we unfortunately can only answer with speculations. Part of this may have to do with the fact that c(3)G, cona, and to a lesser extent corolla have large coiled-coil domains. This is a general feature of the transverse filament (Page and Hawley 2004). As discussed by Kursel et al., the coiled-coil structure may be more

tolerant of amino acid replacements and thus have elevated rates due to relaxed constraints (Kursel et al. 2021). In flies, perhaps the combination of relaxed constraint and positive selection leads to exceptionally elevated rates of protein evolution. Despite having sites under positive selection, lateral elements are not coiled-coils and thus have fewer neutrally evolving sites. With more sites under negative selection, the gene-wide omega will be lower. Whether the difference in rates may also have to do with their interacting partners (chromatin vs other SCs), we think it is possible but find it hard to speculate beyond the fact that the central elements primarily interact with each other. In contrast, the lateral elements will have to interact with conserved proteins. The co-evolution of central elements may therefore be unencumbered with pressure to preserve interactions with conserved proteins. Since there are examples of other meiotic genes directly interacting with the DNA/chromatin that show evidence of rapid evolution, it is unclear whether interaction with DNA/chromatin would necessarily lead to stronger conservation.

Below I outline a few minor comments that I think will help improve the clarity of manuscript.

Minor Comments:

• I got confused between the central elements and the central region—which seems to consist of
both central elements and transverse elements. It would be helpful to be more explicit about this when first introducing the central region components at Line 93.
Now clarified in figure 1A and in the text.

• The molecular evolutionary analyses were conducted across the Drosophila phylogeny and
across key species groups. The combination of the broad-scale and fine-scale analyses is great.
It would be helpful to note how many "evolutionary years" these different groups encompass.
Now added the age of the groups in Figure 1B.

• Figure 1: The Blast% plots are very interesting. I would rather see this analysis for all five SC
genes in the main text and move the Blast% for vtd to the Supplement with the other pairing genes. I understand that the authors are trying to contrast the striking divergence of the SC genes to other pairing genes, however I think this is eOectively done in panel G showing pairwise
Blastp % identity. I understand that moving the 3 additional Blast% heatplots might be challenging in terms of space. In general, I think components of Figure 1 can be smaller without
sacrificing clarity, particularly panel A, the width of the panel B phylogenetic tree, and the

Blast% heatplots could be smaller. Additionally, due to its placement, I initially thought that the
Blast % identity scalebar was specific to panel E instead of to all the heatplots. Perhaps this scale can be moved to the top? Finally, what are the numbers in parentheses in panel G?
We have reworked figure 1 to include the suggestions. The numbers previously in 1G (now 1I) represent lineages with duplications - now clarified in the figure legend.

• Line 129: I understand the authors definition of malformed in text. In Figure 1, the authors represent “malformed” SC genes with a grey box, but all also have a blue symbol indicating a
copy. Does this mean that the SC genes in these species are still malformed? Or were they previously malformed but fixed in this paper by hand-annotation? Can the authors better Clarify?
These malformed annotations were manually fixed. This has now been clarified.

• Figure 2: The authors note that: “unless connected by lines, separate blocks of the same color
represent diOerent locations of the same element”. I found this notation a bit subtle for such an
important result. This finding should be better represented in the text and in the figure. To emphasize this finding, I would rather see Supplementary Figure 6A in this main text figure instead of the dotplots. In general, dotplots might be hard for a broad reader to interpret, I would favor moving them to the Supplement.
We have more strongly emphasized this gene movement results in the text, but elected to keep Figure 2 as is, since the most striking examples of movements are captured by the color scheme in 2A and B. Although dotplots are not the most intuitive plot type, we prefer them in this instance for the clear illustrations of tandem duplications vs transposition. They can also convey sequence similarity and offer a sense of sequence divergence between aligning and adjacent regions. The example of multiple transpositions in *kikkawai* (many of which are fragmented) (Figure 2E) is hard to convey without using the dotplots.

• Line 152 and below is about duplication events. This text should be moved to the section below
that discusses repeated duplications and paralogs.
This paragraph (under the heading “Poor sequence conservation and frequent duplications of components of the SC”) is meant as a general overview of the broad patterns of evolution of SC genes including poor conservation and duplications. The following section (heading: “Complex history of repeated duplication and loss of paralogs”) then discusses the nuance/complexity of the duplications.

• Figure 3: I find the depictions of the Alpha-fold structures to be a bit cramped making it hard to

distinguish which regions correspond to diOerent species.
More space has now been added between the protein structures in 3A and F.

• I understand that Kursel et al. showed that Caenorhabditis SC proteins evolve neutrally, but
without reading the paper, this is a nuanced point in the face of “structural but not sequence conservation”. I wonder if this finding is better communicated to a naïve reader by noting instead that Caenorhabditis SC proteins have conserved coiled-coil domains and near invariant protein length but are among the most diverged sequences in Caenorhabditis.
Now read: “This unique evolutionary property of structural but not sequence conservation is also observed by Kursel et al. in *Caenorhabditis*, whereby central element genes have conserved coiled-coil domains and near invariant protein lengths, while being some of the most divergent proteins.”

• The convergent c(3)G duplications in D. athabasca/D. aOinis and D. triauraria is so striking. This
data needs a slower introduction to drive home this data. In particular, the D. athabasca/D. aOinis and D. triauraria duplications are independent and therefore, should not both be named
the same. Instead of naming both c(3)G2, the authors should choose separate identifiers.
We have now added a preamble (“Even though *D. triauraria c(3)G2* and *D. athabasca/affinis c(3)G2* arose independently and are found on different chromosomes (Figure 2A), ...)” to emphasize the distance of the two species, prefacing the introduction of the convergent evolution of the two independent duplicates. We understand the point regarding nomenclature to denote orthologs/paralogs found in the same vs. different syntenic locations, but this becomes very tricky given how often c(3)G, cona, and corolla move around and are found in non-syntenic regions. Even c(3)G1 - the “primary copies” in both lineages - are found on the different chromosomes in triauraria and athabasca/affinis, and would then have to be somehow designated differently. Instead we elected to make clear their non-syntenic relationships in the text when mentioned as exemplified in the quoted sentence above.

• Independent cona duplicates in D. sechellia and D. serrata should also have diOerent identifiers
(see comment above)
Same reason listed above regarding the difficulty of having separate and consistent identifiers.

• The section on coronetta, while interesting, is a bit long. I suggest that the authors trim this section by removing some of the extraneous details from Hemmer & Blumenstiel and merging a
shortened version of this section into the previous section that outlines novel germline

functions.
This section has been trimmed.

• Line 289: gene-wide omega values are predominantly negative—do the authors mean close to zero?
Yes we did - corrected.

• Figure 5: Is it possible to match the order of the species in Panels A and B to the order of the
species in Figure 2?
We have reoriented the c(3)G tree in figure 5 to match the tree in figure 2.

• How does the expression of SC genes in D. miranda from single cell RNA-seq data compare to
that in D. melanogaster? Do these also have pre-meiotic activity? Presumably Li Zhao's data set
should be able to address this.
According to the flycell atlas, SC genes show the strongest expression in the early primary spermatocytes - now mentioned.

• The Discussion section "Trials & Tribulations can be shortened or entirely removed.
We are electing to keep this section not only for the sake of documentation but also as a guide/warning for the many researchers interested in taking advantage of the wealth of genomic resources of Drosophila species.

• Line 18: awkward sentence, might need a fix grammatically
Corrected the missing word.

• Line 26: awkward sentence, might need a fix grammatically
Reworked the sentence to be less tortured.

• Line 70: SC abbreviation here in introduction used before SC is defined at Line 76
Corrected.

• Lines 155: species spelled wrong
Corrected.

• Line 347: testes should be capitalized
Corrected.

• Line 450: subspecies spelled wrong

Corrected to “species subgroup”.

• D. sechellia spelled wrong throughout
Corrected.

Reviewer #2:

In this manuscript, Zakerzade and Chang et al. examine the evolution and expression of SC components in Drosophila. This is an interesting topic as the SC is essential for meiosis and has a stereotypical appearance on electron micrographs. However, the proteins that make up the SC are not conserved across distant clades. What drives the divergence of SC proteins and the implications for meiosis are important areas of research.

To explore this topic, Zakerzade and Chang et al. manually curate SC genes in a species-dense set of Drosophila genomes and identify several previously undescribed gene duplication events including an ancient paralog of cona in the Drosophila subgenus. The authors examine the rate of evolution of SC genes/proteins using pairwise identity and measures of omega (dN/dS) along branches. They find, as others have previously shown, that SC proteins are highly diverged, and they conclude that this divergence is due to positive selection. They also examine the expression patterns of SC components in testes and ovaries find that the ovaries/testes bias is variable among species. Their main conclusions are that SC genes/proteins have elevated rates of evolution and evolve under positive selection, have diversified by way of gene duplication generating paralogs with novel germline functions, and that the regulation of SC gene expression is also rapidly evolving.

While parts of this manuscript are solid (identification of paralogs, manual curation of genes, and new genomes/transcriptomes sequenced - highlighted below), I ultimately have major concerns about most of the results and subsequent conclusions and cannot support publication.

Here is a non-exhaustive list of major flaws/concerns which I explain in more detail following.

1) The results for tests for positive selection presented in the main text and figures do not match the raw results provided by the authors.

2) The conclusions surrounding gene-wide omega and species-group-specific test of recurrent positive selection rely on “data not shown”

3) Conclusions about rapid regulatory evolution do not have controls

4) The conclusions about novel male germline function are based mostly on expression, which is not a reliable proxy for function

Here I provide more details about my major concerns:

Concern #1: I first noticed that the main text does not match the data presented in Figure 5A and B and Supplementary Figure 13, which display results from branch-specific rates of protein evolution carried out in HyPhy using aBSREL. (ex. line 297 says that 28 branches show positive selection but Fig. 5A has 24 branches labeled as significant for positive selection). So, I decided to look at the raw results files provided by the authors (the .json files). These files clearly state the number of branches significant for positive selection in the “test results” section. Unfortunately, the numbers in the results file do not match the data provided in the figures or what the authors say in the text.

For example, in the results file for c(2)M (c2M.species.CDS.mafft.nt_ali.fasta.ABSREL.json), which corresponds to Fig 5A: "P-value threshold":0.05, "positive test results":6, "tested":91

This very clearly indicates that there were 91 branches tested and 6 were significant for positive selection. The same applies to c(3)G, cona, corolla and ord, which show 6, 4, 10, and 5 branches significant for positive selection in the raw results files, but the authors claim 38, 26, 34 and 24 significant branches, respectively. The authors indicate in the methods that they used a custom Python script to parse the .json files. I sincerely wonder whether the script collected uncorrected p-values instead of those corrected for multiple testing.

As noted in the methods, we elected to report the nominal p-value. This is because based on a power analysis it is quite clear that the multiple testing correction would be overly stringent. In the quantile quantile plot below, we can get a sense of the behaviour of the statistical procedure employed by Hyphy. First, it is not very sensitive and therefore unable to differentiate p-values at the high end (bottom left). Second, we can also observe the issue with multiple testing corrections by contrasting two genes. For the control gene (vtd - a conserved gene), a nominal p-value threshold of 0.05 would yield 3 tests being significant - an expected false negative rate given that 88 branches were tested for the null hypothesis (i.e. there is no positive selection). However, for c(3)G, we can see the observed p-value distribution is substantially elevated compared to the expected. At 106 tests and a nominal p-value threshold of 0.05, 5.3 false positives are expected, but 30 positives are observed. With Bonferroni correction, only 2 would pass and with FDR (used by the program) 13 would pass.

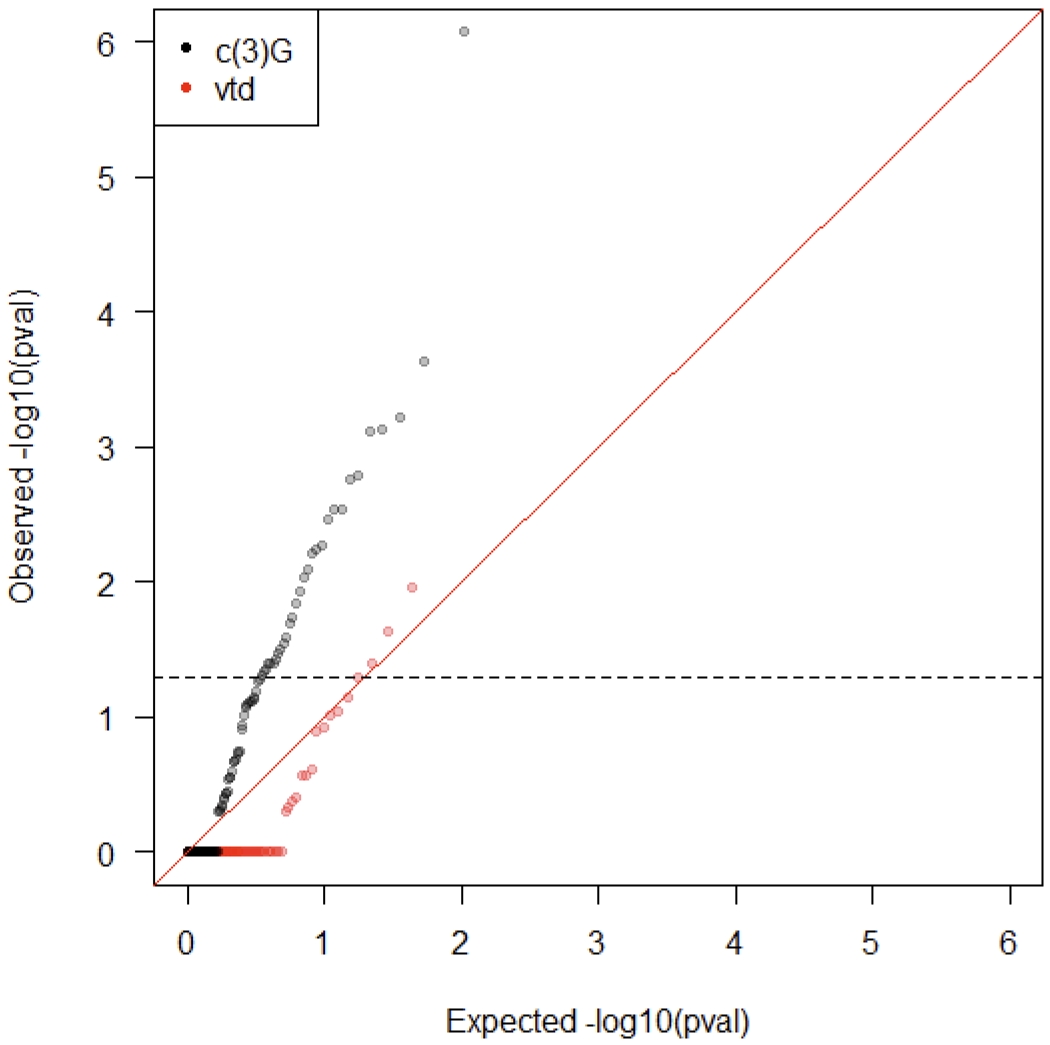

The use of multiple testing correction is always a practice of balancing specificity and sensitivity (Schweder and Spjøtvoll 1982), and in this instance the correction method would remove many true positives. We have now further clarified that nominal p-value is being reported in the results section, and for transparency, we have added Table 1, documenting the number of branches with high omega, and significance with p-value before and after correction for each gene. We also added the above figure as supplementary figure 19 showing the underpowered statistical test by Hyphy, justifying the use of the nominal p-value.

Lastly, our claim that the SC proteins are evolving adaptively is not solely based on the number of significant branches, but instead on a combination of observations including branches with >1 omega, and disproportionately high gene-wide omega compared to all other genes (Figure 5D), and significantly elevated Ka/Ks compared to other meiotic pairing genes (Figure 5C).

Concern #2: The authors say they used PAML and found evidence of positive selection (lines 306 – 308 in the text) but there are no results, no data (other than this statement) and no information in the methods regarding what programs within PAML they used or tests they did. This amounts to “data not shown”, which is unacceptable.

Part of the results is captured in Figure 5D, which we have now correctly labeled. The group-specific PAML results are now in the Supplementary Table 4.

Concern #3: The authors see that some SC genes are expressed higher in the ovaries in one species, and higher in the testes in a closely related sister species. From this they conclude that the SC genes have “poor regulatory conservation”. Without proper controls, this is simply an observation. Does the expression of other germline genes shift between species? Since the author use mostly previously published RNAseq, it should be relatively straightforward to perform the same analysis on all germline enriched genes as a control.

We have added Figure 6B and 6C, showing the expression variability of SC genes in two ways. In 6B, we show the distribution of SC expression and importantly the variance compared to the meiotic pairing genes in testes (ovaries in supplementary figure 15 A). These analyses show that the SC genes generally have more variable expression than pairing genes across species. In 6C, we parsed orthology calls from orthodb and estimated the branch-specific regulatory evolution by first inferring the ancestral expression at internal nodes (using phytools fastanc() function), and then calculated the rate of change at each branch. We then estimated the standard deviation of the branch-specific rates across the SC as well as genes with genome-wide orthology calls in orthodb. Note that we had to discard more than half of the species because they lacked orthology calls in orthodb. This analysis showed that compared to genes highly expressed in ovaries, only ord has conserved expression, while the others rank between 83- 96.4 percentile of genes. For genes highly expressed in testes, SC genes rank between 72-83 percentile. Although not the fastest, this is on the higher end of the distribution for both testes and ovary expression.

Concern #4: It has become widely accepted that expression does not amount to function. In particular, the testis seems to be a tissue with promiscuous gene expression and there are multiple examples of genes with testis-biased expression in Drosophila that do not appear to have a primary function in the testis. The last paragraph of the Discussion section in https://doi.org/10.7554/eLife.71279 summarizes this nicely.

We agree that elevated testes expression may not correspond to primary testes function. But, our observation opens the possibility that these SC genes can have germline function outside of what they are best known for (recombination in female meiosis). The frequent and ancestral testes expression also lines up with the observation that SC proteins localize to the centromere in premeiotic spermatocytes and mutants have subtle defects in premeiotic pairing (Rubin et al. 2022). While the purpose of these activities is still unknown

and appears to have no gross downstream consequences, these cellular phenotypes strongly support that the expression of SC genes, at least in melanogaster, is not just the product of promiscuous transcription. We also want to note that, as described in the paragraph you recommended, even if high testes expression doesn't translate to function in a given species, it can nonetheless precipitate diversification and innovation of the genes as per the out-of-testes hypothesis, which is entirely consistent with our overall thesis. We have now incorporated these possibilities into the discussion.

Valuable parts of the study

1) Manual curation and hand annotation of a large number of SC genes - efforts to scrutinize computerized annotations are often undervalued. But it is clear that, especially for very divergent genes, annotations are frequently wrong, which can result in erroneous conclusions. Therefore, it is important to curate the dataset, as the authors here have done.

2) The careful identification of gene duplication events, including the ancient paralog of cona (called coronetta) – the identification of SC paralogs was thoughtful and careful. The authors took genomic context (synteny) into account and analyzed bootstrap-supported gene trees to make conclusions.

3) Assemblies of D. hypocausta and D. niveifrons and testes and ovaries RNA-seq of eight new species are a valuable contribution to Drosophila genomics.

Reviewer #3:

Zakerzade and Chang et al. take advantage of the plethora of new Drosophilidae sequence data and new data they generate to analyze the patterns of evolution of genes in the synaptonemal complex (SC). Through a painstaking process, they demonstrate that despite being essential for female meiosis, genes in the synaptonemal complex are rapidly evolving in protein sequence, copy-number, and regulation. They show that SC genes are prone to frequent duplication across Drosophilidae, and that these paralogs often follow a similar pattern of structural evolution. Perhaps most interestingly, the authors find that both single copy SC genes and their paralogs are prone to acquiring testes expression. This is strange as meiotic pairing in the Drosophila male germline is achiasmate and doesn't use the synaptonemal complex. This tendency thus indicates that SC genes and their paralogs may play an important and largely uncharacterized role in the male germline.

This work constitutes a major step forward in our understanding of the evolution of the synaptonemal complex. It also sets the stage for future functional studies to better

understand the mysterious role that the SC genes may play in the male germline in D. melanogaster and across Drosophila. Zakerzade and Chang et al. also point out a crucial hurdle for evolutionary genetic studies in Drosophila – gene annotation. They show that despite the hundreds of high-quality Drosophila genomes now available, proper annotation is the rate limiting factor for analyses of rapidly evolving genes, and manual annotation is often still needed.

There are a few aspects of this manuscript that can be improved. Namely, the authors rely on expression data to support the claim that SC genes and their paralogs may have a critical function in the male germline. This may lead to some potentially faulty assumptions. For example, looking at expression alone, the pattern of expression of C(3)G and cona in D. melanogaster (high in testes, low in ovaries) would suggest that it should play a crucial role in male germline development, and a less important role in females. This doesn't seem to be the case. The Rubin et al. (PNAS, 2022) study they cite shows that though they suffer a reduction in efficiency of premeiotic pairing, C(3)G and cona mutant males do not show any defects in chromosome territory formation, chromosome segregation or reduced fertility. The authors should consider discussing explanations for the discrepancy between gene expression data and experimental observations of necessity in D. melanogaster, as this point is crucial for the conclusions they make about a potential male germline function of SC genes across Drosophila.
We have added a discussion regarding the issue of inferring of testes function based on gene expression alone, and that testes expression is known to be promiscuous. Regarding the results from Rubin et al, we have a somewhat different take away. Despite not seeing gross mutant phenotypes as mentioned, they identified subtle and significant cellular phenotypes (such as slower pre-meiotic pairing). To us, this demonstrates a cellular function for SC genes (at least cona and c(3)G) in the testes even if not essential, which lends support to our suggestion that SC genes and their paralogs may also function in testes across the genus. Note we do not make any claims about paralogs active in the testes having critical, necessary, or essential function.

The authors never discuss the expression patterns of SC genes outside of the male and female germline. I wonder if they also see somatic regulatory variability for SC genes, or whether their variability in expression is limited to the germline.
As far as we can tell, there is very little activity outside the gonads (albeit in very restricted numbers of species). We have now added a sentence describing no expression outside of gonads and added Supplementary figure S17, showing that the SC genes have low to no expression in other tissues (based on Flyatlas data displayed on flybase).

Smaller edits:

Sentence ending on line 57 is missing a reference.

Added.

Line 232: Figure 2 C-E seem to have no information on the expression of c(3)G paralogs.
Corrected to 3C-E.

Line 272: It is not immediately clear how Figure 4A demonstrates conservation. It may be that the gene tree is much tighter, but this is hard to see visually.
The branch lengths for conta are so short that you cannot barely make them out. We have now pointed this out in the figure legend.

Line 276: The wrong figure is referenced.
Corrected - should be Supp fig. 12

Line 293: Supplementary figure 12 seems to be the wrong figure.
Corrected - should be Supp fig. 13

Figure 5 legend: Supplementary figure 12 seems to be the wrong figure.
Corrected - should be Supp fig. 13

Line 332: Supplementary figure 12 seems to be the wrong figure.
Corrected - should be Supp fig. 13

Line 418: Supplementary figure 15 seems to be the wrong figure.
Corrected - it is now Supp fig. 18

Line 449-450: The authors use ‘pseudoobscura subspecies.’ This may be misleading, as there are multiple subspecies of Drosophila pseudoobscura, but they are intending to refer a translocation that occurred in the ancestor of D. pseudoobscura, D. persimilis, and D. miranda. Perhaps they should change to ‘pseudoobscura group species.’
Corrected - now reads “pseudoobscura species subgroup”

Perhaps a more comprehensive figure legend would be helpful for supplementary figure 2.
Now reads: “**Supplementary Figure 2:** Dotplots of self-alignments of the 500kb regions on the neo-X (A) and neo-Y (B) containing *ord*. Repeated tandem duplications can be observed generating many alignments off the diagonal.”

Supplementary figure 5 legend is cut off.
Corrected.
