# Supplementary material for: Diversification and recurrent adaptation of the synaptonemal complex in Drosophila
Source: PLoS Genet. 2025 Jan 13;21(1):e1011549. doi: 10.1371/journal.pgen.1011549 (PMC11761671; doi:10.1371/journal.pgen.1011549)
Supplement: Attachment — Submitted filename: replytoeditor.pdf [file pgen.1011549.s027.pdf]

Dear editor,

We appreciate the comments and suggestions by you and the reviewers and have addressed all of the recommended changes. Please see the point-by-point replies below. Raw reads and intermediate files have all been deposited on the SRA and Dryad, respectively, and will be released upon acceptance.

Thank you very much for submitting your revised manuscript "Diversification and recurrent adaptation of the synaptonemal complex in Drosophila”" (PGENETICS-D-24-00785R1) to PLOS Genetics. Your revised manuscript was evaluated by the original three reviewers, and reviewers 1 & 3 were satisfied with the changes.

Reviewer #2 also felt that the revised manuscript was greatly improved, but has a few more minor recommendations they feel are important before publication. I see that the main text now does mention the issue with nominal p-values from the HyPhy test, so I don’t think that needs to be addressed (perhaps the reviewer missed that revision).

We added the sentence below in the relevant Results section:

“we elected to use the nominal p-value as the test is demonstrably underpowered – see Materials and Methods.”

I agree with the reviewer that the use of “consistently” to describe the PAML results (line 331 in the revision) does not reflect the set of P-values in Supplemental Table 4, and that it would be beneficial to move this table to the main text. I also agree that adding the M8 vs. M8a comparison from PAML would be a valuable addition to the analyses.

We have expanded the PAML results into Table 2 which now includes p-values for the M8a vs M8, as well as statistics from the M8 site model (% sites under positive selection and their Omega values). With the exception of *c(2)M* in the melanogaster group, the two comparisons show significance in the same genes and species groups. We would like to note that because the wrapper software PaPAML does not report M8a vs M8, we reran the site model tests using codeml in PAML, which produced slightly lower p-values of M7 vs M8 than in our previous version which has now been updated. While we removed “consistently”, our usage is not merely referring to the significance tests, but also other evidence of positive selection including number of significant branches and the branches with omega greater than 1. As with all significant tests, p-values need to be interpreted with care, especially when we know the tests require a lot of assumptions. It is also worth mentioning that an insignificant p-value should not be used to confirm the null hypothesis. In this instance, while none of tests for *cona* came out significant, large numbers of branches show omega greater than 1; the discrepancy is most likely due to cona being a short gene and therefore has fewer sites for the comparison needed for significance. This is further exacerbated by the fact that high protein evolution will obscure sequence alignment. These PAML results are now more clearly described as below:

“We further used PAML to infer the rate of protein evolution within the three well represented species groups, and we find evidence of group-specific positive selection although not significant in all instances (Table 2). For *c(2)M* and *ord*, the *melanogaster* and *obscura* groups, respectively, show significant group-wide positive selection based on the M7 vs. M8 model comparison, while in branch-specific tests, all species groups have at least one branch that is either significant or has omega > 1. For *c(3)G* and *corolla*, two of the three groups show significance. Lastly, while *cona* does not pass significance in any species group, they all show high numbers of branches with omega > 1. Because *cona* is a short gene of only ~220 AA, tests based on amino acid substitutions are inherently going to be underpowered. Similarly heterogeneous rates of protein evolution across clades have also been observed in rapidly evolving genes and pathways, such as those involved in programmed double strand breaks and DNA repair [51,52].”

Finally, I also agree that in the instances indicated by the reviewer, “expression” is a better word choice than “activity”.

We have made these changes.

I personally also agree that some of the adjectives/adverbs (curiously, surprisingly) are not necessary, but I believe that should be the authors’ decision.

We have made some of the recommended changes as well as others where we see fit.

I had one question regarding Figure 6B. The manuscript text indicates that the expression variance is greater for SC genes than meiotic pairing genes, but unless I misunderstand the Brown–Forsythe test, I assume that the greater variance in SC genes would result in significant P-values for those genes? In Figure 6B the ** indicate it is the pairing genes with significantly different variances. Given that the Brown–Forsythe test is not so commonly used, I think some explanation here might help the reader, particularly if a significant result indicates equal variance.

The Brown–Forsythe test (Brown and Forsythe 1974) determines whether two populations have equal variance and a significant p-value rejects equal variance. In Figure 6B, the stars summarize the significance values of pairwise comparisons between the pairing genes and SC genes (1 star represents significant differences in some comparisons, while two stars represent significant differences in all comparisons; the full pairwise comparisons are represented in Supp figure 15). As usual, significance in-of-itself does not indicate which population has the greater variance; the point-spreads in the boxplots show higher variance for SC genes. We have made further clarification in the figure legends:

“p-value is from the Brown–Forsythe test of equal variance whereby the variances of two populations are compared and equal variance is rejected with a significant test”

And added the below citation:

Brown, Morton B., and Alan B. Forsythe. 1974. “Robust Tests for the Equality of Variances.” *Journal of the American Statistical Association* 69 (346): 364.

I also found a number of little typos and errors, but I probably missed others, so I recommend a careful re-reading of the manuscript before resubmitting.

We have now carefully proof-read the manuscript and corrected typos.

page 8 line 168: “should be previously unaccounted for”

Corrected.

line 237: C(3)G should be c(3)G

Corrected.

line 309: Notedly should be Notably

Corrected.

line 325: what is the word “Note” doing here?

Changed to “We note that”.

line 489: “protein sequences” should be “protein sequence”

Corrected.

Journal Requirements:

Please update "Summary" section to be "Author Summary". This should appear in your manuscript between the Abstract and the Introduction, and should be 150u2013200 words long. The aim should be to make your findings accessible to a wide audience that includes both scientists and non-scientists. Sample summaries can be found on our website under Submission Guidelines:
https://journals.plos.org/plosgenetics/s/submission-guidelines#loc-parts-of-a-submission

Done.

Zakerade *et al.* have addressed most of my major concerns. However, I do have a few comments/requests that I feel strongly should be addressed prior to publication. My underlying concern is that the data are not

over interpreted by the authors (in particular, the tests for positive selection) and are presented in an unbiased manner in the results.

Here are my previous major concerns and my response to the most recent draft of the manuscript. My requests prior to publication are underlined.

1) The results for tests for positive selection presented in the main text and figures do not match the raw results provided by the authors.

The authors explain this discrepancy by noting that they are using uncorrected p-values for the aBSREL analysis because the HyPhy statistical tests are underpowered. They now provide uncorrected and FDR-corrected p-values for this test as well as a power analysis in Supplementary Fig 19. This makes sense to me. **I ask that the authors mention in the main text that they chose to interpret their results with the nominal p-value because test is under powered.**

2) The conclusions surrounding gene-wide omega and species-group-specific test of recurrent positive selection rely on "data not shown"

The authors have added their PAML results as a supplementary table. Now that I can evaluate the results, I have a few requests before the data are published.

- I find the statement in the results about the PAML analysis misleading given what is reported in the table. They state:

  Line 315 "We further used PAML to infer the rate of protein evolution within the three well represented species groups, and we, again, consistently find evidence of group-specific positive selection (Supplementary table 4)."

  When I look at the table, there is not consistent evidence of group-specific positive selection, as the authors state. Instead, some genes show evidence of positive selection in some species groups. For example, *c(3)g* shows evidence of positive selection in the mel and obs groups but not immigrams group. Cona show no evidence of positive selection in any group. **What the authors write in the main text needs to reflect the results in the table and Supplementary Table 4 should be moved to the main text.**

  2) The authors only report results from M7 vs M8 comparison. In M7 (the null hypothesis in this case) omega forced to be less than 1. A better null hypothesis would be M8a, where omega can be less than or equal to 1, allowing true neutral evolution to be included in the null hypothesis. This is especially pertinent for the SC, where others have found that. lack of constraint has contributed to the high divergence of the SC proteins. **I would like the authors to add results for M8a vs M8 prior to publication. If M7 vs M8 is significant, but M8a vs M8 is not, it suggests that neutral evolution drives the diversification of the SC.**

(Copied from response to editor above)

We have expanded the PAML results into Table 2 which now includes p-values for the M8a vs M8, as well as statistics from the M8 site model (% sites under positive selection and their Omega values). With

the exception of *c(2)M* in the melanogaster group, the two comparisons show significance in the same genes and species groups. We would like to note that because the wrapper software PaPAML does not report M8a vs M8, we reran the site model tests using codeml in PAML, which produced slightly lower p-values of M7 vs M8 than in our previous version which has now been updated. While we removed "consistently", our usage is not merely referring to the significance tests, but also other evidence of positive selection including number of significant branches and the branches with omega greater than 1. As with all significant tests, p-values need to be interpreted with care, especially when we know the tests require a lot of assumptions. It is also worth mentioning that an insignificant p-value should not be used to confirm the null hypothesis. In this instance, while none of tests for *cona* came out significant, large numbers of branches show omega greater than 1; the discrepancy is most likely due to cona being a short gene and therefore has fewer sites for the comparison needed for significance. This is further exacerbated by the fact that high protein evolution will obscure sequence alignment. These PAML results are now more clearly described as below:

"We further used PAML to infer the rate of protein evolution within the three well represented species groups, and we find evidence of group-specific positive selection although not significant in all instances (Table 2). For *c(2)M* and *ord*, only the *melanogaster* and *obscura* groups, respectively, show evidences of significant group-wide positive selection based on the M7 vs. M8 model comparisons, while all species groups have at least one branch that is either significant or has omega > 1. For *c(3)G* and *corolla*, two of the three groups show significance. Lastly, while *cona* does not pass significance in any species group, they all show high numbers of branches with omega > 1. Because *cona* is a short gene of only ~220 AA, tests based on amino acid substitutions are inherently going to be underpowered. Similarly heterogeneous rates of protein evolution across clades have also been observed in rapidly evolving genes and pathways, such as those involved in programmed double strand breaks and DNA repair [51,52]."

3) Conclusions about rapid regulatory evolution do not have controls

The authors have added context to the regulatory evolution of the SC genes in Fig 6C. I find this to be a nice control.

4) The conclusions about novel male germline function are based mostly on expression, which is not a reliable proxy for function

The authors have contextualized their findings of male germline expression in the Discussion, which I find helpful. However, I disagree with the use of the word "activity." (ex. Line 360 "high testis activity"). Why not just say high testis expression since this is what the authors are measuring?

Summary Line 19 "germline activities" should be changed to "germline expression patterns".

Changed to "germline expression patterns:

Line 360 "high testis activity" should be changed to "high testis expression"

Changed.

Line 362 “suggesting ancestral testes function” should be changed to “suggesting ancestral testes expression”

Changed.

5) Lastly, a more minor concern. I find some the language and superlatives used by the authors in the results section to be unscientific and contribute to over interpretation of the results. Here are some examples:

Lines 196, 211, 245, 388 “Curiously”

196 – kept

211 – kept

245 – removed

388 – cannot find this instance

Line 203 “surprisingly”

Kept

Lines 233, 239 “remarkably”

233 – kept

236 – removed

Line 254 “Adding to the intrigue”

Kept

Line 365 “striking”

Kept

Line 374, 393 “drastic”

374 – kept

393 – removed

Line 401 Highly

Removed

In all cases, these words could simply be deleted and the results would be reported in a more neutral manner. It’s up to the reader to decide whether the results are drastic, remarkable, surprising or striking.
