# Supplementary material for: Diversification and recurrent adaptation of the synaptonemal complex in Drosophila
Source: PLoS Genet. 2025 Jan 13;21(1):e1011549. doi: 10.1371/journal.pgen.1011549 (PMC11761671; doi:10.1371/journal.pgen.1011549)
Supplement: Attachment — Submitted filename: Zakerzade_PLoSGenetics.docx [file pgen.1011549.s024.docx]

In this manuscript, Zakerzade and Chang *et al.* examine the evolution and expression of SC components in Drosophila. This is an interesting topic as the SC is essential for meiosis and has a stereotypical appearance on electron micrographs. However, the proteins that make up the SC are not conserved across distant clades. What drives the divergence of SC proteins and the implications for meiosis are important areas of research.

To explore this topic, Zakerzade and Chang *et al.* manually curate SC genes in a species-dense set of Drosophila genomes and identify several previously undescribed gene duplication events including an ancient paralog of cona in the Drosophila subgenus. The authors examine the rate of evolution of SC genes/proteins using pairwise identity and measures of omega (dN/dS) along branches. They find, as others have previously shown, that SC proteins are highly diverged, and they conclude that this divergence is due to positive selection. They also examine the expression patterns of SC components in testes and ovaries find that the ovaries/testes bias is variable among species. Their main conclusions are that SC genes/proteins have elevated rates of evolution and evolve under positive selection, have diversified by way of gene duplication generating paralogs with novel germline functions, and that the regulation of SC gene expression is also rapidly evolving.

While parts of this manuscript are solid (identification of paralogs, manual curation of genes, and new genomes/transcriptomes sequenced - highlighted below), I ultimately have major concerns about most of the results and subsequent conclusions and cannot support publication.

Here is a non-exhaustive list of major flaws/concerns which I explain in more detail following.

1) The results for tests for positive selection presented in the main text and figures do not match the raw results provided by the authors.
2) The conclusions surrounding gene-wide omega and species-group-specific test of recurrent positive selection rely on “data not shown”
3) Conclusions about rapid regulatory evolution do not have controls
4) The conclusions about novel male germline function are based mostly on expression, which is not a reliable proxy for function

Here I provide more details about my major concerns:

**Concern #1:** I first noticed that the main text does not match the data presented in Figure 5A and B and Supplementary Figure 13, which display results from branch-specific rates of protein evolution carried out in HyPhy using aBSREL. (ex. line 297 says that 28 branches show positive selection but Fig. 5A has 24 branches labeled as significant for positive selection). So, I decided to look at the raw results files provided by the authors (the .json files). These files clearly state the number of branches significant for positive selection in the “test results” section. Unfortunately, the numbers in the results file do not match the data provided in the figures or what the authors say in the text.

For example, in the results file for c(2)M (c2M.species.CDS.mafft.nt_ali.fasta.ABSREL.json), which corresponds to Fig 5A:

*"P-value threshold":0.05,*

*"positive test results":6,*

*"tested":91*

This very clearly indicates that there were 91 branches tested and 6 were significant for positive selection. The same applies to c(3)G, cona, corolla and ord, which show 6, 4, 10, and 5 branches significant for positive selection in the raw results files, but the authors claim 38, 26, 34 and 24 significant branches, respectively. The authors indicate in the methods that they used a custom Python script to parse the .json files. I sincerely wonder whether the script collected uncorrected p-values instead of those corrected for multiple testing.

**Concern #2:** The authors say they used PAML and found evidence of positive selection (lines 306 – 308 in the text) but there are no results, no data (other than this statement) and no information in the methods regarding what programs within PAML they used or tests they did. This amounts to “data not shown”, which is unacceptable.

**Concern #3:** The authors see that some SC genes are expressed higher in the ovaries in one species, and higher in the testes in a closely related sister species. From this they conclude that the SC genes have “poor regulatory conservation”. Without proper controls, this is simply an observation. Does the expression of other germline genes shift between species? Since the author use mostly previously published RNAseq, it should be relatively straightforward to perform the same analysis on all germline enriched genes as a control.

**Concern #4:** It has become widely accepted that expression does not amount to function. In particular, the testis seems to be a tissue with promiscuous gene expression and there are multiple examples of genes with testis-biased expression in Drosophila that do not appear to have a primary function in the testis. The last paragraph of the Discussion section in <https://doi.org/10.7554/eLife.71279> summarizes this nicely.

Valuable parts of the study

1) Manual curation and hand annotation of a large number of SC genes - efforts to scrutinize computerized annotations are often undervalued. But it is clear that, especially for very divergent genes, annotations are frequently wrong, which can result in erroneous conclusions. Therefore, it is important to curate the dataset, as the authors here have done.
2) The careful identification of gene duplication events, including the ancient paralog of cona (called coronetta) – the identification of SC paralogs was thoughtful and careful. The authors took genomic context (synteny) into account and analyzed bootstrap-supported gene trees to make conclusions.
3) Assemblies of *D. hypocausta* and *D. niveifrons* and testes and ovaries RNA-seq of eight new species are a valuable contribution to Drosophila genomics.
