# Supplementary material for: Diversification and recurrent adaptation of the synaptonemal complex in Drosophila
Source: PLoS Genet. 2025 Jan 13;21(1):e1011549. doi: 10.1371/journal.pgen.1011549 (PMC11761671; doi:10.1371/journal.pgen.1011549)
Supplement: Attachment — Submitted filename: Zakerade_PLoSGenetics_Round2.docx [file pgen.1011549.s026.docx]

Zakerade *et al.* have addressed most of my major concerns. However, I do have a few comments/requests that I feel strongly should be addressed prior to publication. My underlying concern is that the data are not over interpreted by the authors (in particular, the tests for positive selection) and are presented in an unbiased manner in the results.

Here are my previous major concerns and my response to the most recent draft of the manuscript. My requests prior to publication are underlined.

1) The results for tests for positive selection presented in the main text and figures do not match the raw results provided by the authors.

The authors explain this discrepancy by noting that they are using uncorrected p-values for the aBSREL analysis because the HyPhy statistical tests are underpowered. They now provide uncorrected and FDR-corrected p-values for this test as well as a power analysis in Supplementary Fig 19. This makes sense to me. **I ask that the authors mention in the main text that they chose to interpret their results with the nominal p-value because test is under powered.**

2) The conclusions surrounding gene-wide omega and species-group-specific test of recurrent positive selection rely on “data not shown”

The authors have added their PAML results as a supplementary table. Now that I can evaluate the results, I have a few requests before the data are published.

- I find the statement in the results about the PAML analysis misleading given what is reported in the table. They state:

*Line 315 “We further used PAML to infer the rate of protein evolution within the three well represented species groups, and we, again, consistently find evidence of group-specific positive selection (Supplementary table 4).”*

When I look at the table, there is not consistent evidence of group-specific positive selection, as the authors state. Instead, some genes show evidence of positive selection in some species groups. For example, *c(3)g* shows evidence of positive selection in the mel and obs groups but not immigrams group. Cona show no evidence of positive selection in any group. **What the authors write in the main text needs to reflect the results in the table and Supplementary Table 4 should be moved to the main text.**

2) The authors only report results from M7 vs M8 comparison. In M7 (the null hypothesis in this case) omega forced to be less than 1. A better null hypothesis would be M8a, where omega can be less than or equal to 1, allowing true neutral evolution to be included in the null hypothesis. This is especially pertinent for the SC, where others have found that. lack of constraint has contributed to the high divergence of the SC proteins. **I would like the authors to add results for M8a vs M8 prior to publication. If M7 vs M8 is significant, but M8a vs M8 is not, it suggests that neutral evolution drives the diversification of the SC.**

3) Conclusions about rapid regulatory evolution do not have controls

The authors have added context to the regulatory evolution of the SC genes in Fig 6C. I find this to be a nice control.

4) The conclusions about novel male germline function are based mostly on expression, which is not a reliable proxy for function

The authors have contextualized their findings of male germline expression in the Discussion, which I find helpful. However, I disagree with the use of the word “activity.” (ex. Line 360 “high testis activity”). Why not just say high testis expression since this is what the authors are measuring?

**Summary Line 19 “germline activities” should be changed to “germline expression patterns”.**

**Line 360 “high testis activity” should be changed to “high testis expression”**

**Line 362 “suggesting ancestral testes function” should be changed to “suggesting ancestral testes expression”**

5) Lastly, a more minor concern. I find some the language and superlatives used by the authors in the results section to be unscientific and contribute to over interpretation of the results. Here are some examples:

Lines 196, 211, 245, 388 “Curiously”

Line 203 “surprisingly”

Lines 233, 239 “remarkably”

Line 254 “Adding to the intrigue”

Line 365 “striking”

Line 374, 393 “drastic”

Line 401 Highly

In all cases, these words could simply be deleted and the results would be reported in a more neutral manner. It’s up to the reader to decide whether the results are drastic, remarkable, surprising or striking.
